# Genome-Wide Association Study Identifies Chromosome 10q24.32 Variants Associated with Arsenic Metabolism and Toxicity Phenotypes in Bangladesh

**DOI:** 10.1371/journal.pgen.1002522

**Published:** 2012-02-23

**Authors:** Brandon L. Pierce, Muhammad G. Kibriya, Lin Tong, Farzana Jasmine, Maria Argos, Shantanu Roy, Rachelle Paul-Brutus, Ronald Rahaman, Muhammad Rakibuz-Zaman, Faruque Parvez, Alauddin Ahmed, Iftekhar Quasem, Samar K. Hore, Shafiul Alam, Tariqul Islam, Vesna Slavkovich, Mary V. Gamble, Md Yunus, Mahfuzar Rahman, John A. Baron, Joseph H. Graziano, Habibul Ahsan

**Affiliations:** 1 Department of Health Studies, The University of Chicago, Chicago, Illinois, United States of America; 2 Comprehensive Cancer Center, The University of Chicago, Chicago, Illinois, United States of America; 3 Columbia University and University of Chicago Research Office in Bangladesh, Dhaka, Bangladesh; 4 Department of Environmental Health Sciences, Mailman School of Public Health, Columbia University, New York, New York, United States of America; 5 International Center for Diarrheal Disease Research, Dhaka, Bangladesh; 6 Lineberger Comprehensive Cancer Center, University of North Carolina, Chapel Hill, North Carolina, United States of America; 7 Departments of Medicine and Human Genetics, The University of Chicago, Chicago, Illinois, United States of America; University of Oxford, United Kingdom

## Abstract

Arsenic contamination of drinking water is a major public health issue in many countries, increasing risk for a wide array of diseases, including cancer. There is inter-individual variation in arsenic metabolism efficiency and susceptibility to arsenic toxicity; however, the basis of this variation is not well understood. Here, we have performed the first genome-wide association study (GWAS) of arsenic-related metabolism and toxicity phenotypes to improve our understanding of the mechanisms by which arsenic affects health. Using data on urinary arsenic metabolite concentrations and approximately 300,000 genome-wide single nucleotide polymorphisms (SNPs) for 1,313 arsenic-exposed Bangladeshi individuals, we identified genome-wide significant association signals (P<5×10^−8^) for percentages of both monomethylarsonic acid (MMA) and dimethylarsinic acid (DMA) near the AS3MT gene (arsenite methyltransferase; 10q24.32), with five genetic variants showing independent associations. In a follow-up analysis of 1,085 individuals with arsenic-induced premalignant skin lesions (the classical sign of arsenic toxicity) and 1,794 controls, we show that one of these five variants (rs9527) is also associated with skin lesion risk (P = 0.0005). Using a subset of individuals with prospectively measured arsenic (n = 769), we show that rs9527 interacts with arsenic to influence incident skin lesion risk (P = 0.01). Expression quantitative trait locus (eQTL) analyses of genome-wide expression data from 950 individual's lymphocyte RNA suggest that several of our lead SNPs represent cis-eQTLs for AS3MT (P = 10^−12^) and neighboring gene C10orf32 (P = 10^−44^), which are involved in C10orf32-AS3MT read-through transcription. This is the largest and most comprehensive genomic investigation of arsenic metabolism and toxicity to date, the only GWAS of any arsenic-related trait, and the first study to implicate 10q24.32 variants in both arsenic metabolism and arsenical skin lesion risk. The observed patterns of associations suggest that MMA% and DMA% have distinct genetic determinants and support the hypothesis that DMA is the less toxic of these two methylated arsenic species. These results have potential translational implications for the prevention and treatment of arsenic-associated toxicities worldwide.

## Author Summary

Exposure to arsenic through drinking water is a serious public health issue in many countries, including Bangladesh and the United States. Although there is substantial inter-individual variation in arsenic metabolism and toxicity, the biological basis of this variation is not well understood. Here, we have conducted the first genome-wide association study of arsenic-related traits within a unique population cohort of arsenic-exposed Bangladeshi individuals. Using data on 1,313 well-characterized individuals, we identify multiple association signals for urinary arsenic metabolite concentrations in the 10q24.32 regions, near the AS3MT (arsenite methyltransferase) gene. In a subsequent analysis of >2,000 individuals, we show for the first time that variants that influence arsenic metabolism can also influence risk for arsenical skin lesions (the classical sign of arsenic toxicity) through interaction with arsenic exposure. Using array-based genome-wide gene expression data, we show that several of our lead genetic variants are associated with expression of AS3MT and neighboring gene C10orf32, providing a potential mechanism by which 10q24.32 variants influence arsenic metabolism and toxicity. Knowledge of variation in this region and associated biological processes could be used to develop intervention and pharmacological strategies aimed at preventing large numbers of arsenic-related deaths in arsenic-exposed populations.

## Introduction

Over 100 million individuals worldwide are exposed to arsenic through drinking water, including approximately 56 million people in Bangladesh [1] and 13 million in the United States [2]. Arsenic is a class I human carcinogen, and chronic exposure to high levels of arsenic (>300 µg/L) is associated with substantial increased risk for a wide array of diseases including cancers of the lung [3], bladder [4], liver [5], skin [6], and kidney [7], [8], as well as neurological [9], [10] and cardiovascular [11] diseases. Emerging evidence suggests that arsenic may have adverse effects on health even at concentrations as low as 10–50 µg/L, as recent studies in Bangladesh have observed dose-response relationships with mortality [12], [13] and arsenical skin lesion risk [14] in populations with low to moderate arsenic exposure over many years. Arsenical skin lesions are a classical sign of arsenic toxicity, an indicator of susceptibility to arsenic-related disease, and a precursor to arsenic-induced skin cancers [6]. Once individuals are chronically exposed to arsenic, risk for arsenic-related diseases and mortality remains high for several decades even after cessation of exposure [15], [16].

Consumed arsenic enters the blood as As^V^ and As^III^, known collectively as inorganic arsenic (iAs). Once consumed, iAs is methylated using S-Adenosyl methionine (SAM) as the methyl donor, producing monomethylarsonic acid (MMA) and then dimethylarsinic acid (DMA). MMA is believed to be the more toxic of these metabolites, with the DMA/MMA ratio showing an inverse association with arsenic toxicity in several studies [17]–[20] and DMA being more readily excreted in urine and expelled from the body. Arsenic metabolite concentrations are often expressed as percentages of all arsenic species present in urine (i.e., iAs%, MMA%, DMA%) or as ratios that reflect methylation efficiency (e.g., DMA%/MMA%, MMA%/iAs%).

There is considerable inter-individual variation in arsenic metabolism, as some individuals are able to methylate, and thus excrete, arsenic more efficiently than others [21], [22]. Similarly, because high inter-individual variability in toxicity is observed among individuals with similar levels of exposure to arsenic, genetic susceptibility factors for arsenical skin lesions are believed to exist [23].

In light of the enormous global health impact of arsenic exposure and the remarkable inter-individual variability in arsenic metabolism and toxicity, we performed the first genome-wide association study (GWAS) of common arsenic-related phenotypes. We identified multiple genetic variants in the 10q24.32 region near AS3MT (arsenite methyltransferase, previously known as CYT19) that show robust associations with urinary concentrations of arsenic metabolites, risk for arsenical skin lesions, and local gene expression, including transcript levels of AS3MT.

## Results

### GWAS of arsenic metabolites

We assessed genome-wide associations for the three arsenic metabolites measured in urine (iAs%, MMA%, and DMA%) using high-quality data on 259,597 single-nucleotide polymorphisms (SNPs) from 1,313 individuals randomly selected from a large population-based cohort of Bangladeshi individuals exposed to a wide range of arsenic concentrations through drinking water. Associations were assessed using mixed linear models [24] to account for existence of related individuals in our sample (Figure S1). The strongest association signals, genome-wide, for both DMA% and MMA% were in the 10q24.32 region (P<5×10^−8^) (Figures S2 and S3), which contains the AS3MT gene and substantial LD spanning ∼1 Mb (Figure S4).

**Figure 1:**
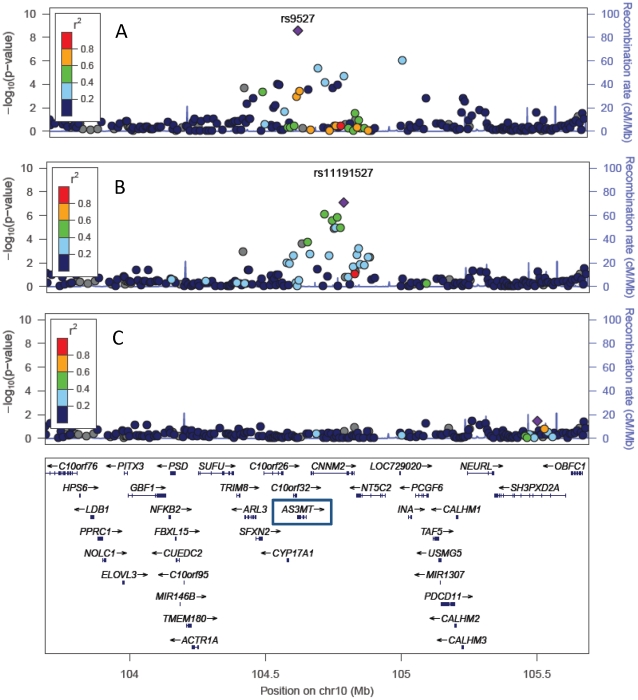
Multiple variants in the 10q24.32 region show independent associations with DMA% (n = 1,313). P-values were generated using mixed models adjusted for age, sex, and water arsenic concentration. The strongest associated SNP is labeled in each panel. Panel A shows the overall association results. Panel B shows P-values from models that are adjusted for rs9527. Panel C shows P-values from models adjusted for both rs9527 and rs11191527.

For DMA%, the strongest 10q24.32 association was for rs9527 (P = 2.7×10^−9^; Figure 1). After conditioning on rs9527, a strong residual association signal remained (rs11191527; P = 8.0×10^−8^), the strength of which was weaker without adjustment for rs9527 (P = 2.3×10^−5^) due to mild LD between these SNPs (D′ = 0.26, r^2^ = 0.03 in our data; D′ = 0.27; r^2^ = 0.03 in HapMap GIH). After conditioning on both SNPs, there was very little evidence of additional association in the region. Analyses of imputed and measured genotypes produced the same two association signals, but with imputed SNPs rs3740394 and rs17115073 showing slightly stronger association than rs9527 and rs11191527, respectively (Figure S5).

**Figure 2:**
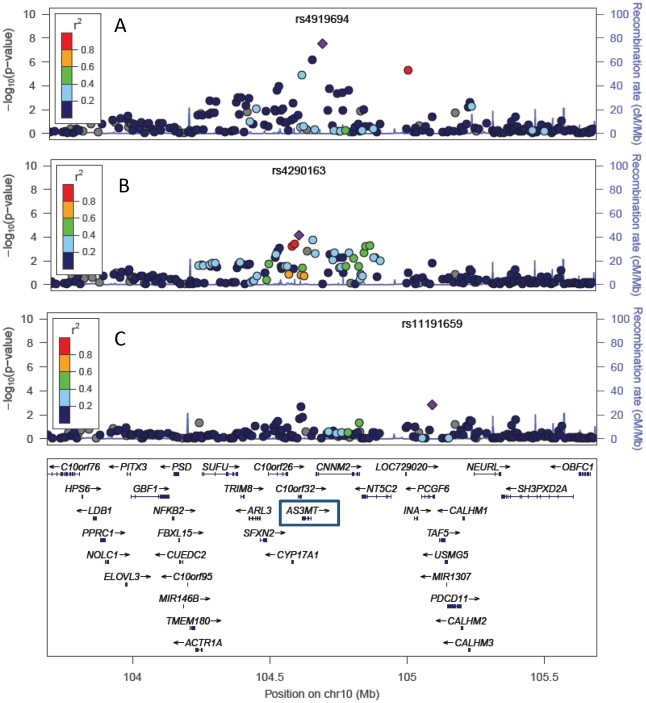
Multiple variants in the 10q24.32 region show independent associations with MMA% (n = 1,313). P-values were generated using mixed models adjusted for age, sex, and water arsenic concentration. The strongest associated SNP is labeled in each panel. Panel A shows the overall association results. Panel B shows P-values from models that are adjusted for rs4919694. Panel C shows P-values from models adjusted for both rs4919694 and rs4290163.

The strongest association observed for MMA% was rs4919694 (P = 2.9×10^−8^) (Figure 2). After conditioning on rs4919694, residual association was still observed (rs4290163; P = 7.0×10^−5^). This association is much weaker without adjustment for rs4919694 (P = 0.03) due to LD between rs4919694 and rs4290163 (D′ = 0.80, r^2^ = 0.09 in our data; D′ = 0.80, r^2^ = 0.04 in HapMap GIH). Aftern conditioning on both SNPs, residual association was observed for rs11191659 (P = 0.0009), a SNP in moderate LD with rs9527, the top SNP from the %DMA analysis (D′ = 0.66, r^2^ = 0.23 in our data; D′ = 0.82, r^2^ = 0.30 in HapMap GIH). Conditioning on all three SNPs eliminated the 10q24.32 association signal. Imputation of unobserved genotypes in the region did not reveal associations stronger than those observed for the measured genotypes (Figure S6). Multivariate models for %DMA and %MMA including all five of the above-mentioned SNPs are described in Table 1. Outside of the 10q24.32 region, there was no genome-wide significant (P<5×10^−8^) association signal for DMA% or MMA%.

**Table 1:** Multivariate associations between arsenic metabolites and genotyped SNPs in the 10q24.32 region showing the strongest univariate associations with DMA% and MMA% (n = 1,313).

		DMA%		MMA%	
SNP (MA^a^)	MAF^b^	Beta coefficient	P-value	Beta coefficient	P-value
rs9527 (A)	0.09	−2.95	0.0002	0.86	0.05
rs11191527 (A)	0.16	2.16	4.4×10^−5^	−0.55	0.06
rs4919694 (G)	0.10	−0.93	0.14	1.49	3.1×10^−5^
rs4290163 (A)	0.43	0.26	0.50	−0.65	0.003
rs11191659 (A)	0.05	−2.07	0.02	1.08	0.04

Regression models including all five SNPs in a single model, adjusting for age, sex, and water arsenic. All regressions were mixed models carried out using EMMAX.

a MA, minor allele.

b MAF, minor allele frequency.

The 10q24.32 association results for the DMA%/MMA% ratio (the “secondary methylation index”, log-transformed), were very similar to the MMA% results, as these phenotypes were strongly correlated (r = −0.84; Table S1). Associations for 10q24.32 SNPs with iAs% and MMA%/iAs% (the “primary methylation index” (PMI) log-transformed) were much weaker than for DMA% and MMA%; The strongest association in the 10q24.32 region observed for iAs% was rs9527 (P = 0.0009) and no association of P<0.001 was observed for log(PMI). In genome-wide analyses of iAs% and PMI, no SNP reached genome-wide significance (Figure S7).

### Association of SNPs with skin lesions risk

Because variants influencing arsenic metabolism may alter susceptibility to arsenic toxicity, we investigated the roles of metabolite-associated SNPs in arsenic-induced premalignant skin lesions, the hallmark of chronic arsenic toxicity. For our five lead SNPs, we tested association with skin lesion status among 1,085 skin lesion cases and 1,794 population controls, using the ROADTRIPS method that was developed for case-control association testing in the presence of cryptic relatedness [25]. The rs9527 allele associated with decreased DMA% (A) was associated with increased skin lesion risk (P = 0.0005), consistent with the hypothesis that DMA is less toxic than MMA (Table 2). rs11191659 showed suggestive association (P = 0.02), also consistent with this hypothesis.

**Table 2:** Association between the 10q24.32 genotyped variants and arsenical skin lesion risk and SNP-arsenic interaction estimates.

		MAF		Logistic regression^b^			ROADTRIPS^c^	Interaction with arsenic^d^	
SNP (MA^a^)	Association with arsenic metabolite	Cases(n = 1,085)	Controls(n = 1,794)	OR	CI	P-value	P-value	Water arsenic P-value	Urine arsenic P-value
rs9527 (A)	↓DMA%	0.108	0.076	1.42	1.16–1.72	0.0005	0.0005	0.004	0.02
rs11191527 (A)	↑DMA%	0.152	0.163	0.96	0.89–1.29	0.38	0.33	0.72	0.38
rs4919694 (G)	↑MMA%	0.103	0.098	1.07	0.89–1.29	0.46	0.60	0.87	0.34
rs4290163 (A)	↓MMA%	0.427	0.434	0.96	0.82–.112	0.59	0.62	0.99	0.23
rs11191659 (A)	↑MMA%	0.058	0.042	1.32	1.02–1.72	0.04	0.02	0.001	<0.0001

a MA, minor allele.

b Each Logistic Regression model includes one SNP, adjusting for age and sex.

c The ROADTRIPS case-control test does not allow multivariate modeling (i.e., no adjustments), but accounts for cryptic relatedness.

d Interaction P-values are from mixed linear models that account for relatedness among subjects. Interactions are on the additive scale and are calculated using data on 69 cases and incident 700 controls.

To confirm that these associations with skin lesions were due to gene-arsenic interaction, we tested the interaction between rs9527 and arsenic exposure using a subset of 69 incident skin lesion cases and 700 controls with prospectively-measured arsenic exposure (measured in both water and urine at baseline, prior to skin lesion incidence and arsenic mitigation efforts [26]). We found SNP-arsenic interaction for both rs9527 (multiplicative interaction P = 0.01; additive interaction P = 0.004) and rs11191659 (multiplicative interaction P = 0.02; additive interaction P = 0.001), where water arsenic exposure showed stronger association with skin lesions in the presence of the risk allele (Table 2 and Table S2). There were no significant main effects for either of these SNPs in the context of models that included SNP-arsenic interaction terms.

For the subset of individuals with available genotype, arsenic metabolite, and skin lesion data (82 cases, 1211 controls), DMA% showed evidence of partial mediation of the association between rs9527 and skin lesions (accounting for 13% of the observed association).

### eQTL analyses of the 10q24.32 region

**Figure 3:**
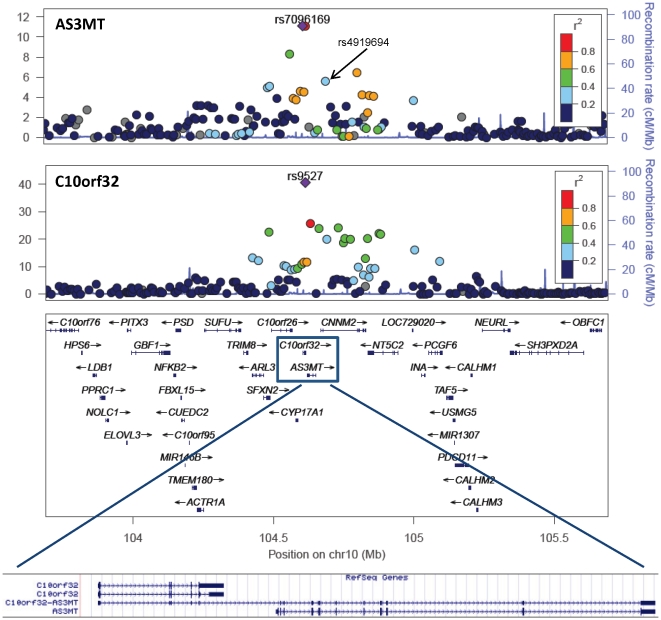
Variants in the 10q24.32 region are associated with transcript levels of AS3MT and C10orf32. C10orf32 is ∼4 kb from AS3MT and involved in C10orf32-AS3MT read-through transcription. P-values were generated using mixed linear models adjusted for age and sex.

To investigate the role of our lead SNPs in gene regulation, used genome-wide expression data derived from lymphocyte RNA obtained at baseline for 950 participants (Illumina HumanHT-12 array) and examined SNP-expression associations for all 30 genes in the 10q24.32 LD region (Table S3). Several of our lead SNPs showed association with AS3MT expression at P<5×10^−5^ (rs4919694, rs9527, rs4290163). However, after examining associations for all SNPs in this region, C10orf32 intronic SNP rs7096169 showed the strongest association with AS3MT expression (P = 8×10^−12^; Figure 3 and Figure S8), and conditioning on rs7096169 eliminated the eQTL signal. rs7096169 was not one of our lead SNPs, but it was associated with DMA% (P = 0.001; MMA% P = 0.28). Interestingly, the rs9527 risk allele (A) was associated with decreased C10orf32 expression (P = 2.6×10^−41^; Figure S8), the strongest eQTL signal for C10orf32 expression in the region (Figure 3) and the strongest genome-wide eQTL effect for rs9527 (Figure S9). C10orf32 is ∼4 kb upstream of AS3MT, and these genes are involved in C10orf32-AS3MT read-through transcription, producing a transcript that is a candidate for nonsense-mediated mRNA decay. Thus, it is possible that the eQTL signal observed for C10orf32 represents a regulatory mechanism that influences read-through transcript production. After conditioning on rs9527, the residual eQTL signal was best represented by rs11083790 (P = 10^−5^). Conditioning on both SNPs eliminated the eQTL signal. Interestingly, C10orf32 expression was also associated with arsenic exposure (measured as total arsenic in urine, collected at the same time as blood; P = 0.001), while AS3MT expression was not (P = 0.37). None of our lead SNPs modified the association between arsenic exposure and C10orf32 expression.

Our lead SNPs were also associated with USMG5 expression (Table S3), a gene ∼500 kb downstream of AS3MT, but these associations appear to be due to moderate LD with downstream variants showing very strong association with USMG5 expression (e.g., rs12220267; P = 10^−210^; Figure S8).

## Discussion

The role of AS3MT in arsenic metabolism has been described [27], and several prior studies have evaluated associations between candidate AS3MT variants arsenic-related traits in Bangladesh and elsewhere [28]–[34]. A recent review [35] highlighted two AS3MT SNPs, rs11191439 (Met287Thr) and rs3740393 (intronic), as being consistently related to arsenic metabolism across diverse populations. The most recent and comprehensive Bangladeshi study of AS3MT SNPs [28] reported three association signals for arsenic metabolites, best represented by HapMap3 SNPs rs1046778 (for MMA%), rs11191439 (DMA% and iAs%), and rs3740390 (DMA% and iAs%), a proxy for rs3740393 (r^2^ = 0.91). After imputation, we were able to replicate rs11191439 (DMA% P = 4.2×10^−6^; MMA% P = 5.8×10^−7^) and rs1046778 (MMA% P = 8.9×10^−7^; DMA% P = 0.0002), which were strongly correlated with lead SNPs rs4919694 (r^2^ = 0.69) and rs4290163 (r^2^ = 0.63), respectively. After conditioning on our lead SNPs, these associations were no longer significant. The evidence for rs3740390 was less convincing (DMA% P = 0.54; MMA% P = 0.007), as this SNP was not strongly correlated with any of our lead SNPs (Figure S10). We identified two novel 10q24.32 association signals, represented by rs9527 and rs11191527, which were not strongly correlated with any previously-reported SNP (Figure S10). These SNPs were likely missed in prior studies due to limited coverage of the SNPs in this region.

The identities of the functional variants in this region remain unclear. rs9527 lies in the 5′ UTR of C10orf32, a transcription factor binding region (GATA-1 and TAL1 (SC-12984)) and a DNase hypersensitivity site. If causal, rs9527 could also exert its effects through regulation of AS3MT-C10orf32 read-through transcription. However, the LD block represented by rs9527 includes transcription factor binding site SNP rs12416687 and miRNA SNPs rs11191401, rs12573077, rs7904252, and rs9527. Detailed information on potential functional variants from HapMap3 (GIH) for each of the 5 SNPs identified is contained in Tables S4, S5, S6, S7, S8. However, genetic variation in this population has not been comprehensively characterized (especially rare variation), and the underlying functional variants may not be present in HapMap3. It is also possible that the underlying causal variants have implications for surrounding genes. For example, rs4919694 and rs11191527 are intronic SNPs within the CNNM2 gene, which is involved in magnesium reabsorption by the kidney [36]. It is possible that magnesium and iAs interact [37], [38], influencing the amount of free arsenic available for methylation.

To our knowledge, this study is the largest genetic association study of arsenic metabolites to date, the only GWAS of arsenic-related traits, the first study to implicate 10q24.32 SNPs in both arsenic metabolism and arsenical skin lesion risk, and one of the earliest GWAS conducted in the developing country setting. Our results suggest that MMA% and DMA% have distinct genetic determinants and highlight the importance of conditional analyses, as LD among alleles with opposing effects can mask associations in univariate analyses. The associations observed in this study are likely due to the effects of unmeasured, potentially rare variants in LD with the measured SNPs and/or substantial allelic heterogeneity, whereby multiple 10q24.32 variants influence arsenic metabolism.

Considering the substantial LD in this region [39], the variation in allele frequencies and LD patterns among the various arsenic-exposed populations under study [40], and the apparent allelic heterogeneity with respect to arsenic metabolism, future DNA sequencing studies are needed to help identify causal variants in the 10q24.32 region. Identifying these variants will help clarify the links between the association signals observed for %DMA, %MMA, and AS3MT/C10orf32 expression. These association signals appear largely independent in our dataset, but perhaps there are underlying causal variants that influence all of these phenotypes. Developing a better understanding the effects of functional variation related to AS3MT will also provide a more nuanced understanding of the biology of arsenic methylation, which can in turn help us better understand how variation in methylation efficiency affects health. Finally, knowledge of this causal variation and the methylation processes that they influence could potentially be exploited for intervention strategies that aim to prevent large numbers of deaths arsenic-exposed populations, by defining susceptibility subgroups and exploiting the biological processes uncovered by genomics for developing pharmacological treatments.

## Materials and Methods

### Study descriptions

The DNA samples genotyped in this study were obtained at baseline recruitment from individuals participating in one of the following studies: The Health Effects of Arsenic Longitudinal Study (HEALS) [41] or the Bangladesh Vitamin E and Selenium Trial (BEST) [42]. GWAS analyses of arsenic metabolites were conducted using urinary arsenic metabolite and SNP data on 1,313 individuals randomly selected from the HEALS study. Analyses of skin lesion data were conducted using genotype data from 1,085 skin lesion cases and 1,794 controls drawn from both studies, including the 1,313 HEALS individuals with metabolite data. Skin lesion cases included individuals with keratosis, melanosis, and leukomelanosis. Gene expression analyses were based on lymphocyte RNA extracted at baseline recruitment for the first 950 BEST participants. A summary of these overlapping sets of samples is provided in Figure S11.

The *Health Effects of Arsenic Longitudinal Study* (HEALS [41]) is a prospective investigation of health outcomes associated with arsenic exposure through drinking water in a cohort of adults in Araihazar, Bangladesh, a rural area east of the capital city, Dhaka. Between October 2000 and May 2002, we recruited healthy married individuals (age 18–75 years) who were residents of the study area for at least five years and primarily consumed drinking water from a local well. We enumerated 65,876 individuals residing in Araihazar, from which we identified a sampling frame of 14,828 eligible residents. Of these 14,828 individuals, 11,746 men and women were enrolled. During 2006–2008, additional recruitment of 8,287 participants from the same underlying source population expanded the cohort size to over 20,000 individuals. All 5,966 wells in the study area were tested for arsenic using graphite furnace atomic absorption spectrometry and individuals reported the primary well from which they drank. At baseline, trained study physicians, blinded to the arsenic measurements, conducted in-person interviews and clinical evaluations and collected spot urine and blood samples from participants in their homes using structured protocols. Similar in-person follow-up interviews were conducted biennially for the entire cohort during the following periods: follow-up 1 during September 2002 to May 2004, follow-up 2 during June 2004 to August 2006, and follow-up 3 during January 2007 to February 2009. At baseline and each follow-up interview, a structured protocol was used to ascertain skin lesions by the study physicians, who had undergone training for the detection and diagnosis of skin lesions [43]. The study protocol was approved by the Institutional Review Boards of The University of Chicago, Columbia University, and the Bangladesh Medical Research Council. Informed consent was obtained from all participants.

The *Bangladesh Vitamin E and Selenium Trial* (BEST) is a 2×2 factorial randomized chemoprevention trial evaluating the long-term effects of vitamin E and selenium supplementation on non-melanoma skin cancer (NMSC) risk. BEST participants are residents of Araihazar (the same geographic area as HEALS participants with 132 overlapping participants), Matlab, and surrounding areas. BEST uses many of the same study protocols as does HEALS, especially arsenic exposure assessment and biospecimen collection protocols. All participants were required to have existing arsenic-related skin lesions to be eligible. A total of 7,000 individuals have been randomized to one of the four treatment arms: vitamin E only (100 IU/day), *L*-selenomethionine only (200 µg/day), both vitamin E and selenium, and placebo. Participants have been actively followed for 6 years and systematic ascertainment of histopathologically-confirmed NMSC has been conducted (including BCC and SCC). For all participants, biological samples, including all fractions of blood including DNA and RNA, urine, toenails, and tumor samples have been collected at baseline, along with clinical and covariate data, creating a biological and data repository that is available for research purposes. The study protocol was approved by the Ethical Review Committee of International Center for Diarrheal Disease Research, Bangladesh, the Bangladesh Medical Research Council, and the Institutional Review Boards of The University of Chicago and Columbia University. Informed consent was obtained from all participants.

In each study, urinary arsenic was measured using graphite furnace atomic absorption spectrometry in a single laboratory [44]. Urinary creatinine was measured by a colorimetric diagnostics kit (Sigma, St Louis, MO, USA). Total urinary arsenic concentration was divided by creatinine to obtain creatinine-adjusted total arsenic concentration (µg/g creatinine) [45]. Urinary arsenic metabolites (arsenobetaine, arsenocholine, arsenite, arsenate, monomethylarseonous acid, and dimethylarsenic acid) were distinguished as described by Ahsan et al. [17], using a high-performance liquid chromatography method for separation of arsenic metabolites, followed by detection using inductively coupled plasma-mass spectrometry with dynamic reaction cell. The percentage of iAs, MMA and DMA in total arsenic was calculated after subtracting asenobetaine and arsenocholine (i.e., nontoxic organic arsenic from dietary sources). Because these metabolites lie on the same biological pathway and are expressed as a percentage of arsenic species, their values show substantial correlation (Table S1).

### Genotyping and quality control

For BEST samples, DNA extraction was carried out from the whole blood using the QIAamp 96 DNA Blood Kit (cat # 51161) from Qiagen, Valencia, USA. For HEALS samples, DNA was extracted from clot blood using Flexigene DNA kit (Cat # 51204) from Qiagen. Concentration and quality of all extracted DNA were checked by Nanodrop 1000. As starting material, 250 ng of DNA was used on the Illumina Infinium HD SNP array according to Illumina's protocol. Samples were processed on HumanCytoSNP-12 v2.1 chips with 299,140 markers and read on the BeadArray Reader. Image data was processed in BeadStudio software to generate genotype calls.

Prior to genotype QC, our genotype data consisted of 2,920 samples typed for 299,140 SNPs. First, we removed DNA samples with very poor call rates (<90%; n = 8) and SNPs that were poorly called (<90%) or monomorphic (n = 39,276). Individuals with gender mismatches were removed (n = 10), as were technical replicate DNA samples run to assure high genotyping accuracy (n = 21). No individuals had outlying autosomal heterozygosity or inbreeding values. After inspecting distributions of SNP and samples call rates, we excluded samples with call rates <97% (n = 2) and SNPs with call rates <95% (n = 103). SNPs with HWE p-values<10^−7^ were excluded (n = 164). This QC resulted in 2,879 individuals with high-quality genotype data for 259,597 SNPs. All QC was performed using PLINK [46].

### Gene expression

RNA was extracted from mononuclear cells preserved in buffer RLT, stored at −86°C using RNeasy Micro Kit (cat# 74004) from Qiagen, Valencia, USA. Concentration and quality of all extracted RNA were checked on Nanodrop 1000. cRNA synthesis was done from 250 ng of RNA using Illumina TotalPrep 96 RNA Amplification kit. As recommended by Illumina we used 750 ng of cRNA on HumanHT-12-v4 for gene expression. The chip contains a total of 47,231 probes covering 31,335 genes.

### Statistical analyses and software tools

Pair-wise kinship coefficients were estimated using PLINK [46] and their distribution is shown in Figure S1. To assess population structure that was unrelated to the relative pairs present in our dataset, we removed one individual from each related pair (kinship coefficient >0.05) and assessed population structure in this dataset of 403 individuals using principal components analysis as implemented in EIGENSTRAT [47]. We found very little evidence of population stratification (Figure S12), with the eigenvalues from the first ten principle components being between 1.123 and 1.184. All SNP association tests for urinary metabolites were conducted using a mixed model that accounted for cryptic relatedness as implemented in EMMAX [24] (rather than principle components), adjusting for water arsenic, sex, and age. All regional association plots were generated using LocusZoom [48]. Association testing for skin lesion status was conducting using PLINK [46] and the ROADTRIPS [25] software developed for case-control association testing in samples with unknown population and pedigree structure. We conducted local imputation for the 10q24.32 region using MACH, the GIH reference panel, and imputation parameters suggested by the developers [49]. The estimated genotype and allele error rates were 0.034 and 0.017, respectively. LD structure in the 10q24.32 region was visualized using Haploview [50]. Information on the potential functional consequences of SNPs in the 10q24.32 regions was obtained using the NIEHS's SNPinfo Web Server [51]. Interaction analyses was conducted using only HEALS incident cases (n = 69) and controls (n = 701). For BEST participants and some HEALS participants arsenic exposure (based on water and urine) was not measured prior to arsenic mitigation efforts [26], so the measured exposure status for these individuals is not likely to reflect long-term arsenic exposure status. Interactions were tested using the SAS 9.2 PROC MIXED procedure, using the “bn” matrix derived using EMMAX. To assess mediation of the association between SNPs and skin lesions, we used the “proportion explained” (PE) equation for odds ratios (PE_OR_ = (OR*_xy_*−OR*_xy|m_*)/(OR*_xy_*−1) where x is an exposure, y is a binary outcome, and m is a potential mediating factor [52]). Genome-wide eQTL analysis for our five lead SNPs was performed using the significance of microarray method as implemented in BRB Array Tools. Promising eQTL effects were then examined using EMMAX as described above, treating the expression values as a quantitative trait. In a similar fashion, arsenic exposure was tested for association with expression traits of interest and for interaction with SNPs in relation to expression traits using PROC MIXED.

## Supporting Information

Figure S1 Distribution of all pair-wise kinship coefficients among 2,879 Bangladeshi individuals with measured genome-wide SNP data. Kinship values are truncated at 0.05. The observed clusters of observations centered at 0.5 0.25, and 0.125 represent full siblings or parent-offspring, half siblings, and, and first cousins pairs, respectively. One individual from each pair of twins or duplicate samples (kinship coefficient of 1.0) was removed from the analysis dataset.
(TIF)

Figure S2 GWAS Results for DMA% (including Manhattan plot, QQ plot, and the strongest associated SNPs).
(TIF)

Figure S3 GWAS Results for MMA% (including Manhattan plot, QQ plot, and the strongest associated SNPs).
(TIF)

Figure S4 Summary of linkage disequilibrium (LD) in the 10q24.32 region. LD data for all SNPs in the GIH HapMap3 panel are shown above (A) and the data for SNPs typed in this study are shown below (B). Dark red squares represent a D′ value near 1 and white squares represent a D′ value near zero.
(TIF)

Figure S5 DMA% associations results for imputed and genotyped SNPs in the 10q24.32 region (n = 1,313). P-values were generated using mixed-models adjusted for age, sex, and water arsenic concentration. The strongest associated SNP is labeled in each panel. The top panel shows the overall association results. The second panel shows P-values from models that are adjusted for rs3740394. The third panel shows P-values from models adjusted for both rs3740394 and rs17115073.
(TIF)

Figure S6 MMA% associations results for imputed and genotyped SNPs in the 10q24.32 region (n = 1,313). P-values were generated using mixed-models adjusted for age, sex, and water arsenic concentration. The strongest associated SNP is labeled in each panel. The top panel shows the overall association results. The second panel shows P-values from models that are adjusted for rs4919694. The third panel shows P-values from models adjusted for both rs4919694 and rs4290163.
(TIF)

Figure S7 Q-Q plots for genome-wide association scans of the iAs% and the primary methylation index (PMI = MMA%/iAs%). Results are based on 1,310 samples and 259,597 SNPs. The EMMAX model is adjusted for sex and water arsenic.
(TIF)

Figure S8 Cis-eQTL signals in the 10q24.32 region. Expression values for AS3MT, C10orf32, and USMG5 are shown by the minor allele count for rs7096160, rs9527, and rs12220267, respectively. Mean expression values are shown as dotted lines.
(TIF)

Figure S9 Association for our 5 lead SNPs with genome-wide transcript levels. Genome-wide eQTL analysis was performed using the Significance of Microarray method as implemented in BRB Array Tools. The first and second most strongly associated transcripts (in red) are C10orf32 and USMG5, respectively. Both are in the 10q24.32 region.
(TIF)

Figure S10 Linkage Disequilibrium between lead SNPs and previously reported variants. Our lead SNPs are shown in boxes and the variants representing the signals previously reported in a Bangladeshi study (Engstrom et al. [30]) are shown in circles.
(TIF)

Figure S11 An overview of the participants and samples used in this work.
(TIF)

Figure S12 Scatter plot of the first two principle components for 403 unrelated study participants (no pair-wise kinship value>0.05).
(TIF)

Table S1 Pair-wise correlations among the arsenic-related urinary phenotypes examined in this study (n = 1,333).
(DOCX)

Table S2 Regression models for interaction between arsenic exposure and rs9257 in relation to skin lesion risk (69 skin lesion cases, 701 controls).
(DOCX)

Table S3 P-values from association test for our 5 lead 10q24.32 SNPs and expression values for all genes in the 10q24.32 LD region (n = 950 individuals).
(DOCX)

Table S4 Functional information for SNPs in LD with rs9527.
(PDF)

Table S5 Functional information for SNPs in LD with rs11191527.
(PDF)

Table S6 Functional information for SNPs in LD with rs4919694.
(PDF)

Table S7 Functional information for SNPs in LD with rs3740394.
(PDF)

Table S8 Functional information for SNPs in LD with rs11191659.
(PDF)
