# Supplementary figures and images for: Genome-Wide Association Study Identifies Chromosome 10q24.32 Variants Associated with Arsenic Metabolism and Toxicity Phenotypes in Bangladesh
Source: PLoS Genet. 2012 Feb 23;8(2):e1002522. doi: 10.1371/journal.pgen.1002522 (PMC3285587; doi:10.1371/journal.pgen.1002522)

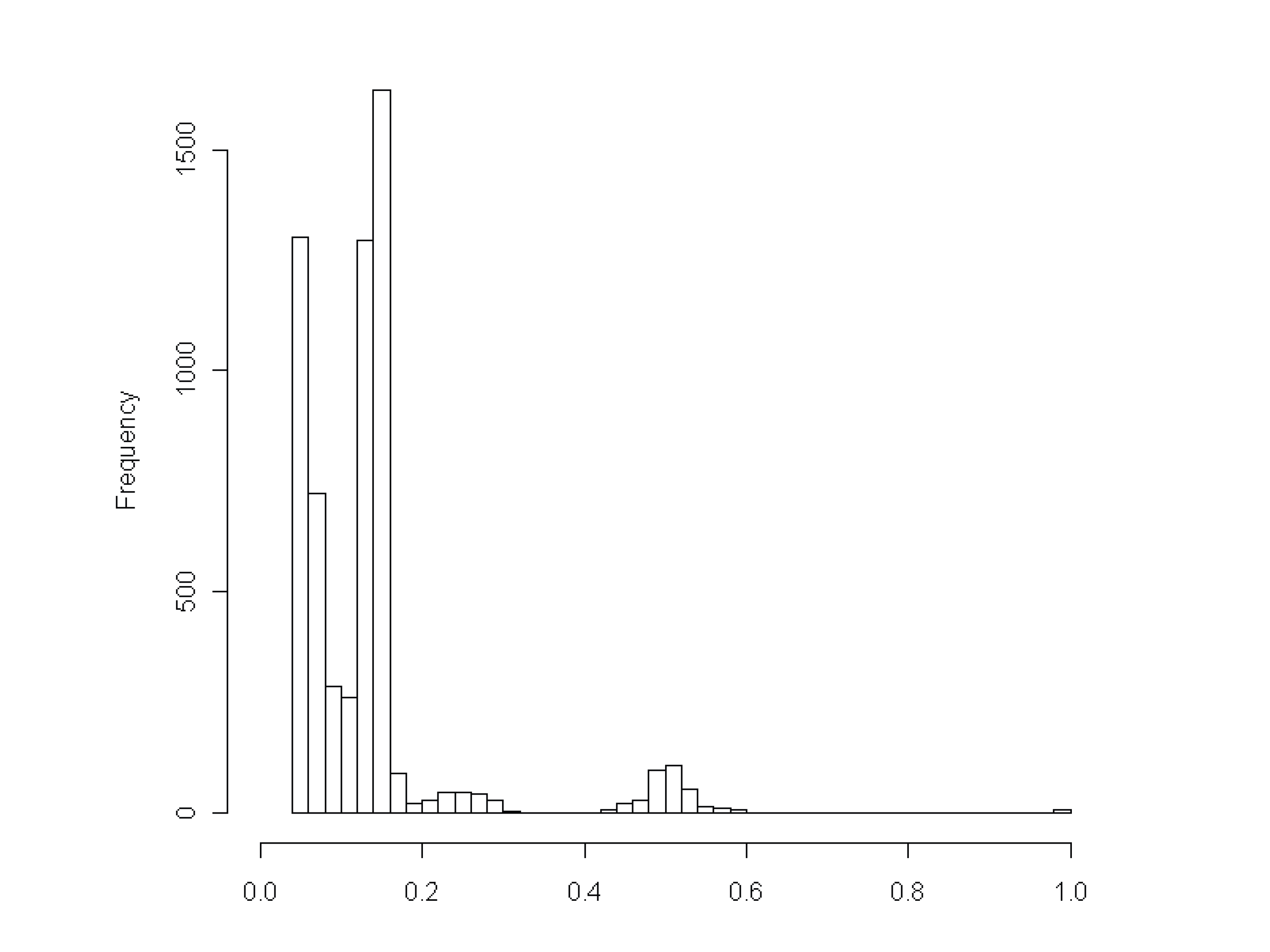

Supplement: Figure S1 — Distribution of all pair-wise kinship coefficients among 2,879 Bangladeshi individuals with measured genome-wide SNP data. Kinship values are truncated at 0.05. The observed clusters of observations centered at 0.5 0.25, and 0.125 represent full siblings or parent-offspring, half siblings, and, and first cousins pairs, respectively. One individual from each pair of twins or duplicate samples (kinship coefficient of 1.0) was removed from the analysis dataset. (TIF) [file pgen.1002522.s001.tif]

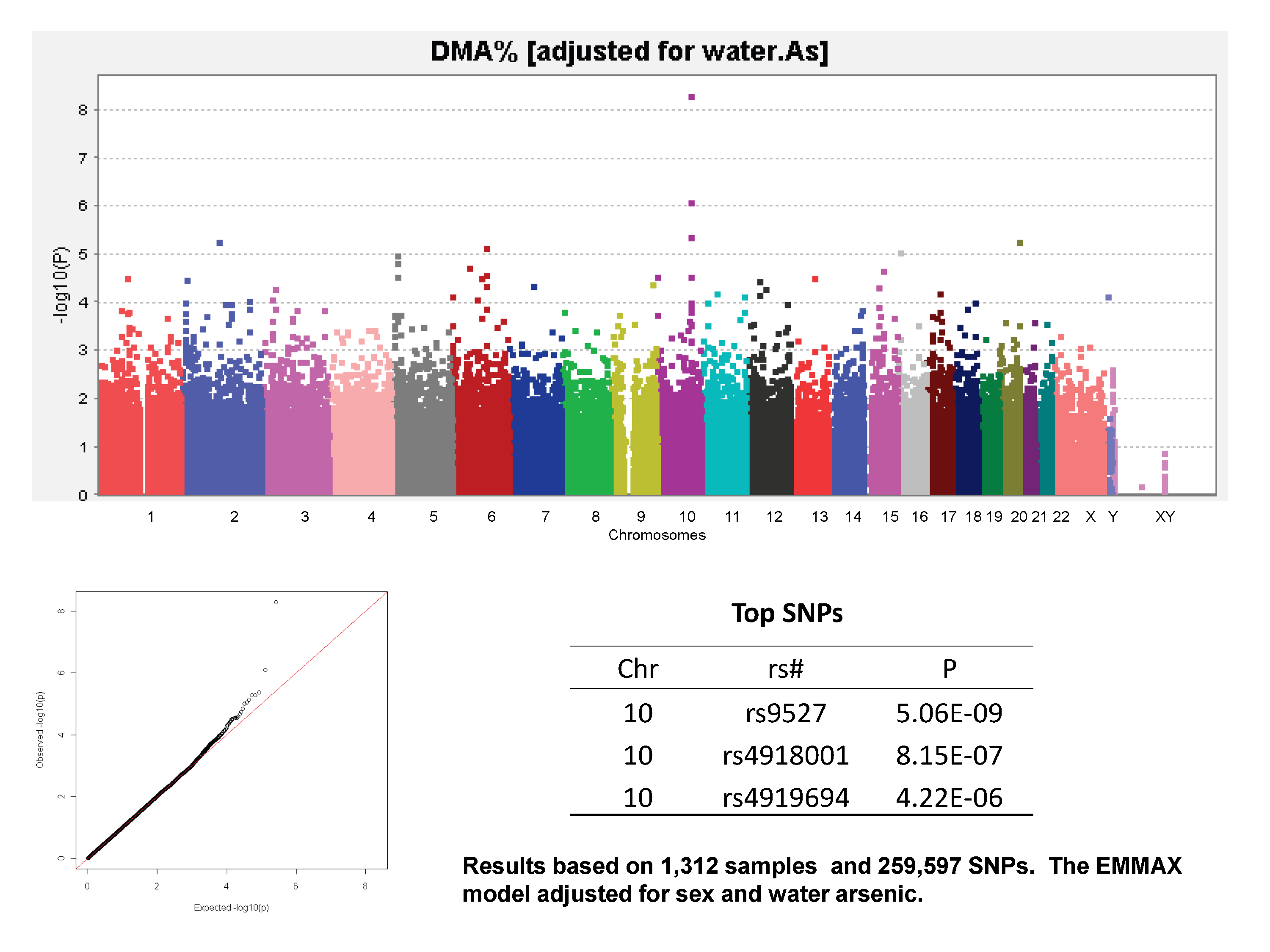

Supplement: Figure S2 — GWAS Results for DMA% (including Manhattan plot, QQ plot, and the strongest associated SNPs). (TIF) [file pgen.1002522.s002.tif]

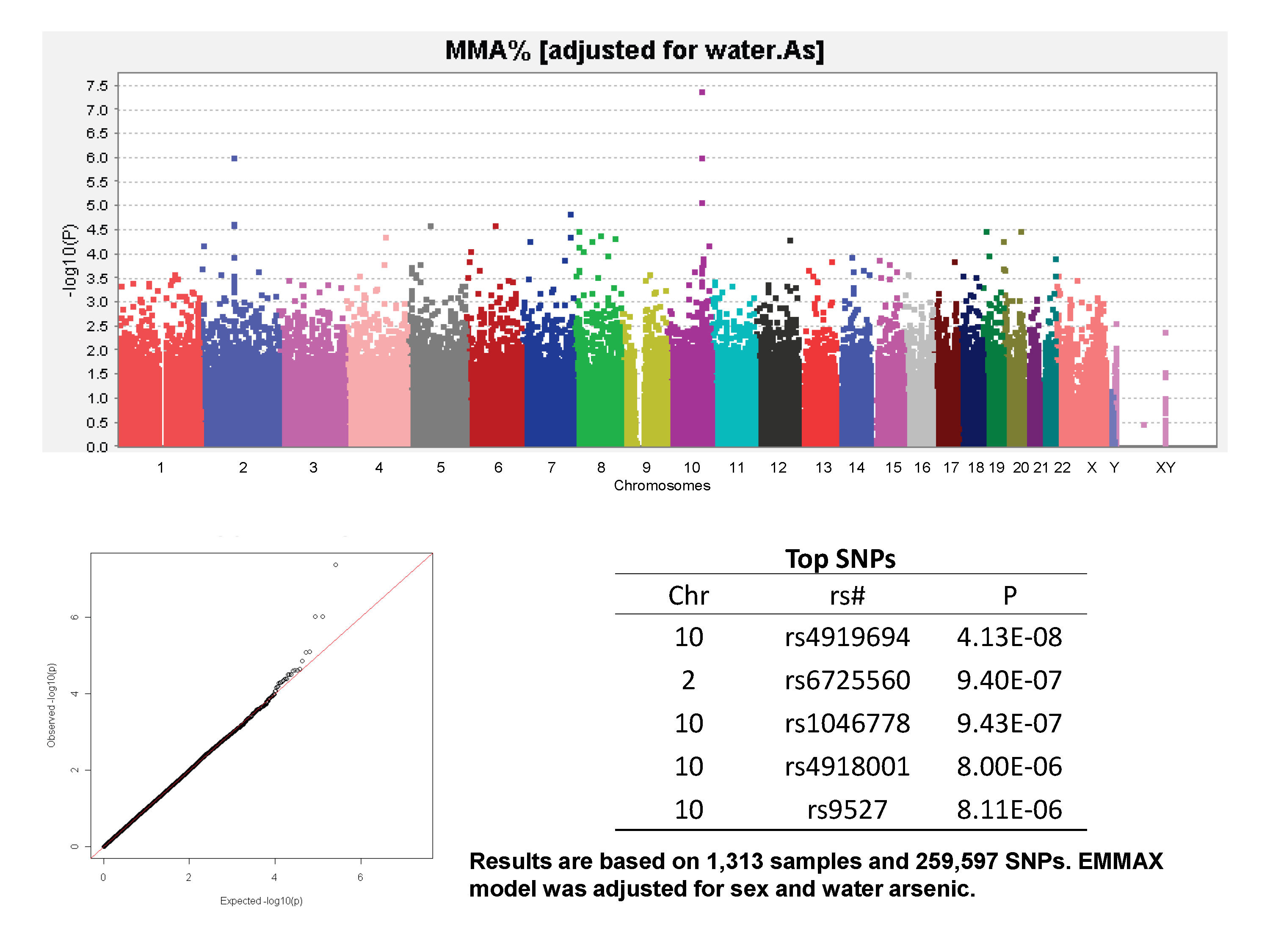

Supplement: Figure S3 — GWAS Results for MMA% (including Manhattan plot, QQ plot, and the strongest associated SNPs). (TIF) [file pgen.1002522.s003.tif]

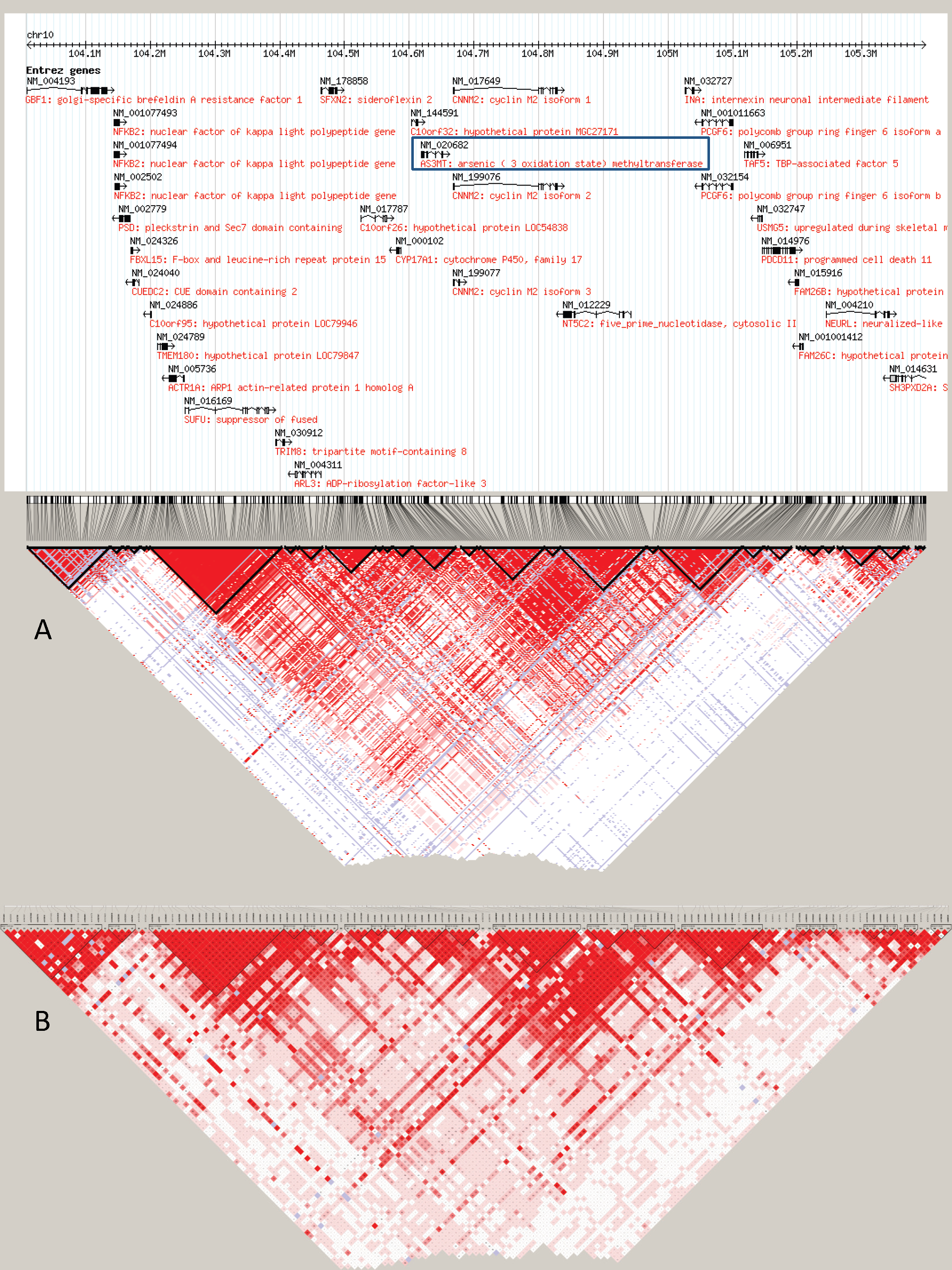

Supplement: Figure S4 — Summary of linkage disequilibrium (LD) in the 10q24.32 region. LD data for all SNPs in the GIH HapMap3 panel are shown above (A) and the data for SNPs typed in this study are shown below (B). Dark red squares represent a D′ value near 1 and white squares represent a D′ value near zero. (TIF) [file pgen.1002522.s004.tif]

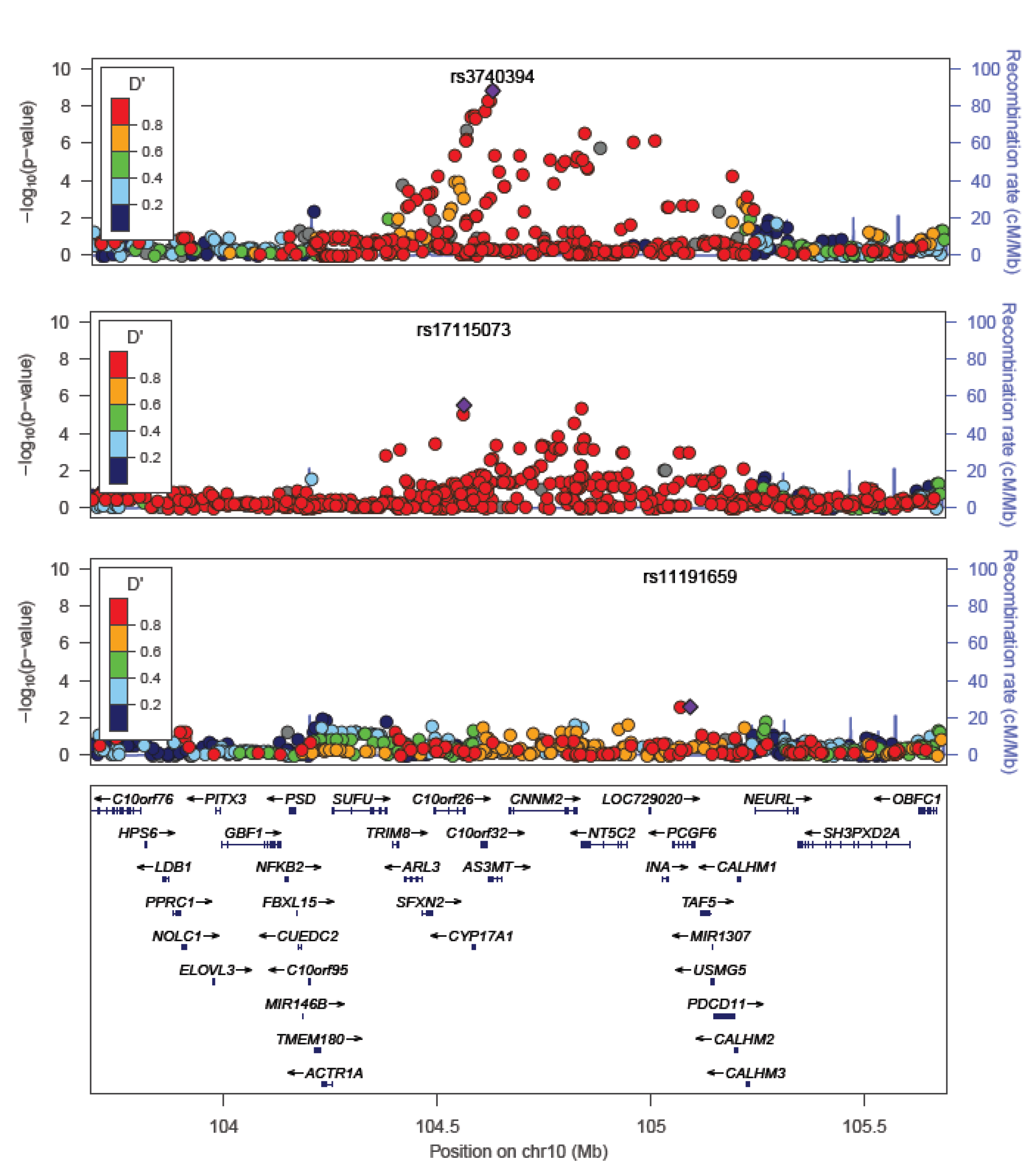

Supplement: Figure S5 — DMA% associations results for imputed and genotyped SNPs in the 10q24.32 region (n = 1,313). P-values were generated using mixed-models adjusted for age, sex, and water arsenic concentration. The strongest associated SNP is labeled in each panel. The top panel shows the overall association results. The second panel shows P-values from models that are adjusted for rs3740394. The third panel shows P-values from models adjusted for both rs3740394 and rs17115073. (TIF) [file pgen.1002522.s005.tif]

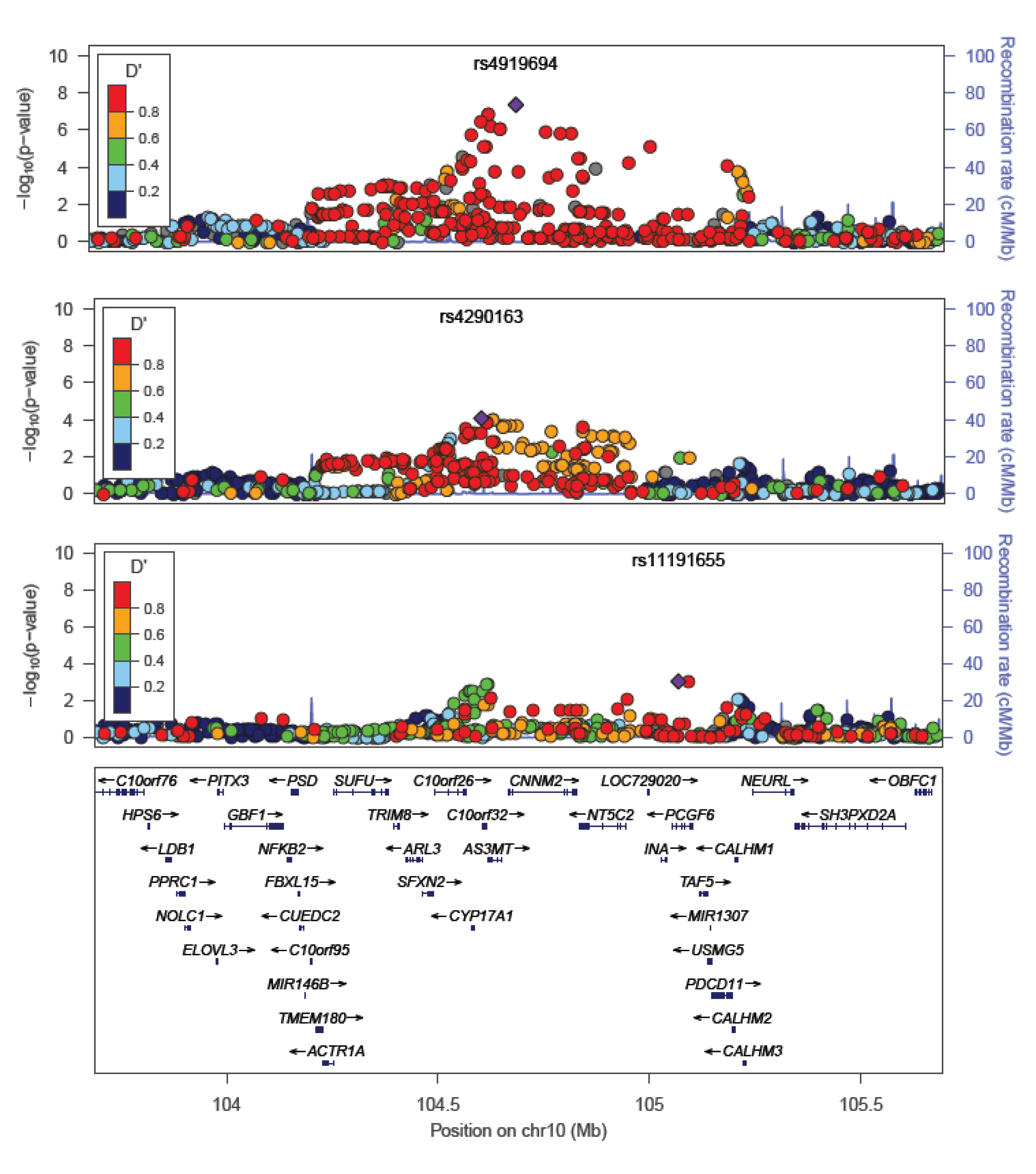

Supplement: Figure S6 — MMA% associations results for imputed and genotyped SNPs in the 10q24.32 region (n = 1,313). P-values were generated using mixed-models adjusted for age, sex, and water arsenic concentration. The strongest associated SNP is labeled in each panel. The top panel shows the overall association results. The second panel shows P-values from models that are adjusted for rs4919694. The third panel shows P-values from models adjusted for both rs4919694 and rs4290163. (TIF) [file pgen.1002522.s006.tif]

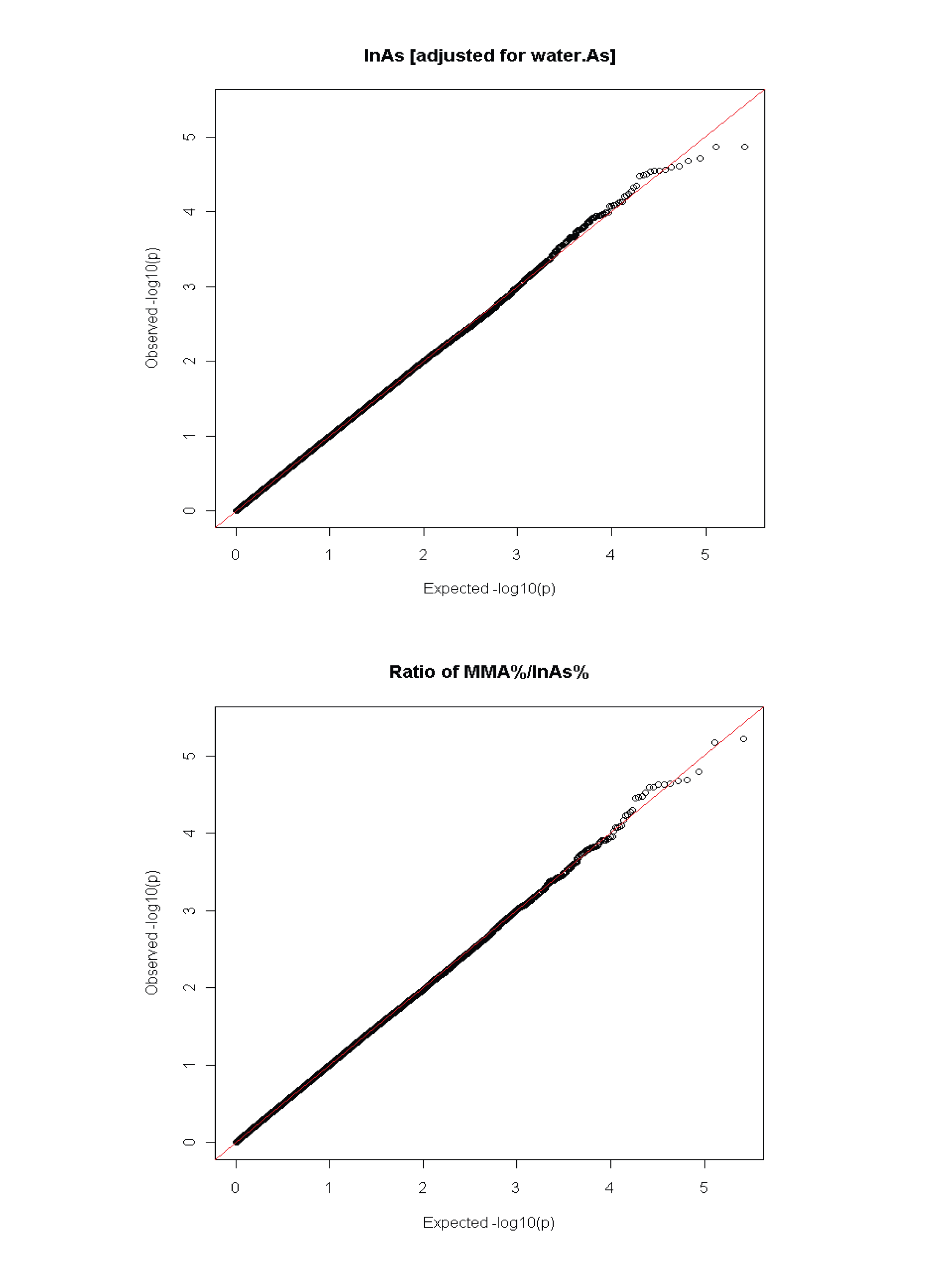

Supplement: Figure S7 — Q-Q plots for genome-wide association scans of the iAs% and the primary methylation index (PMI = MMA%/iAs%). Results are based on 1,310 samples and 259,597 SNPs. The EMMAX model is adjusted for sex and water arsenic. (TIF) [file pgen.1002522.s007.tif]

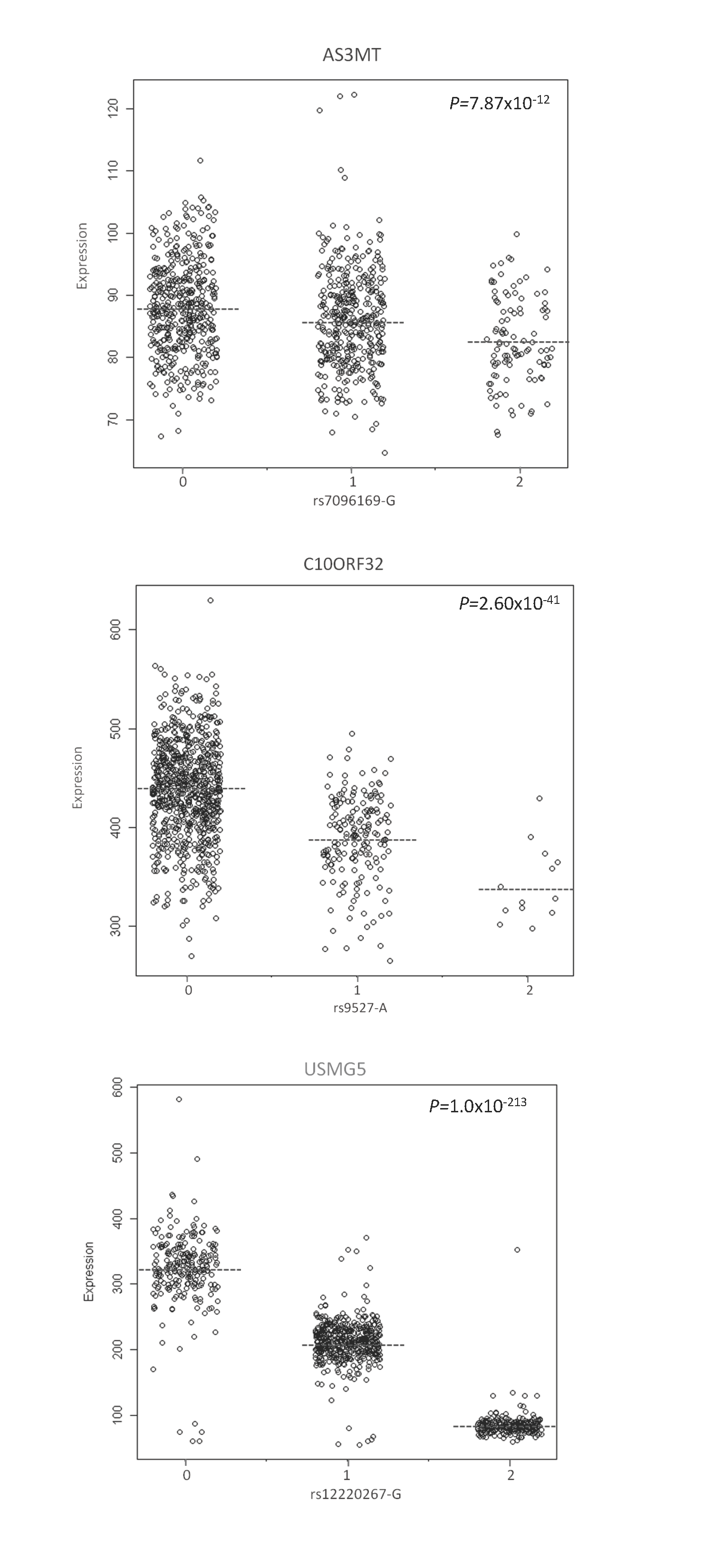

Supplement: Figure S8 — Cis-eQTL signals in the 10q24.32 region. Expression values for AS3MT, C10orf32, and USMG5 are shown by the minor allele count for rs7096160, rs9527, and rs12220267, respectively. Mean expression values are shown as dotted lines. (TIF) [file pgen.1002522.s008.tif]

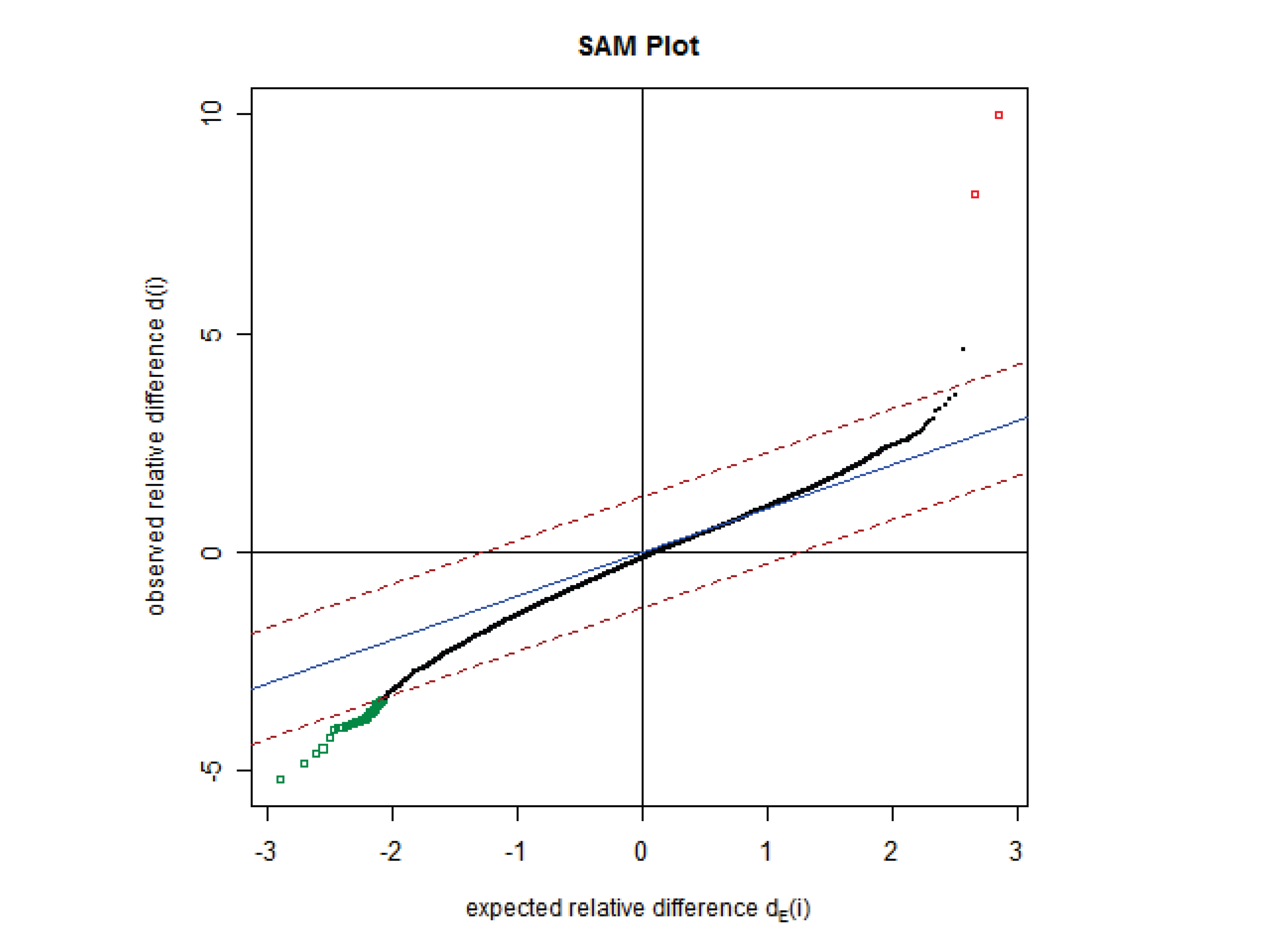

Supplement: Figure S9 — Association for our 5 lead SNPs with genome-wide transcript levels. Genome-wide eQTL analysis was performed using the Significance of Microarray method as implemented in BRB Array Tools. The first and second most strongly associated transcripts (in red) are C10orf32 and USMG5, respectively. Both are in the 10q24.32 region. (TIF) [file pgen.1002522.s009.tif]

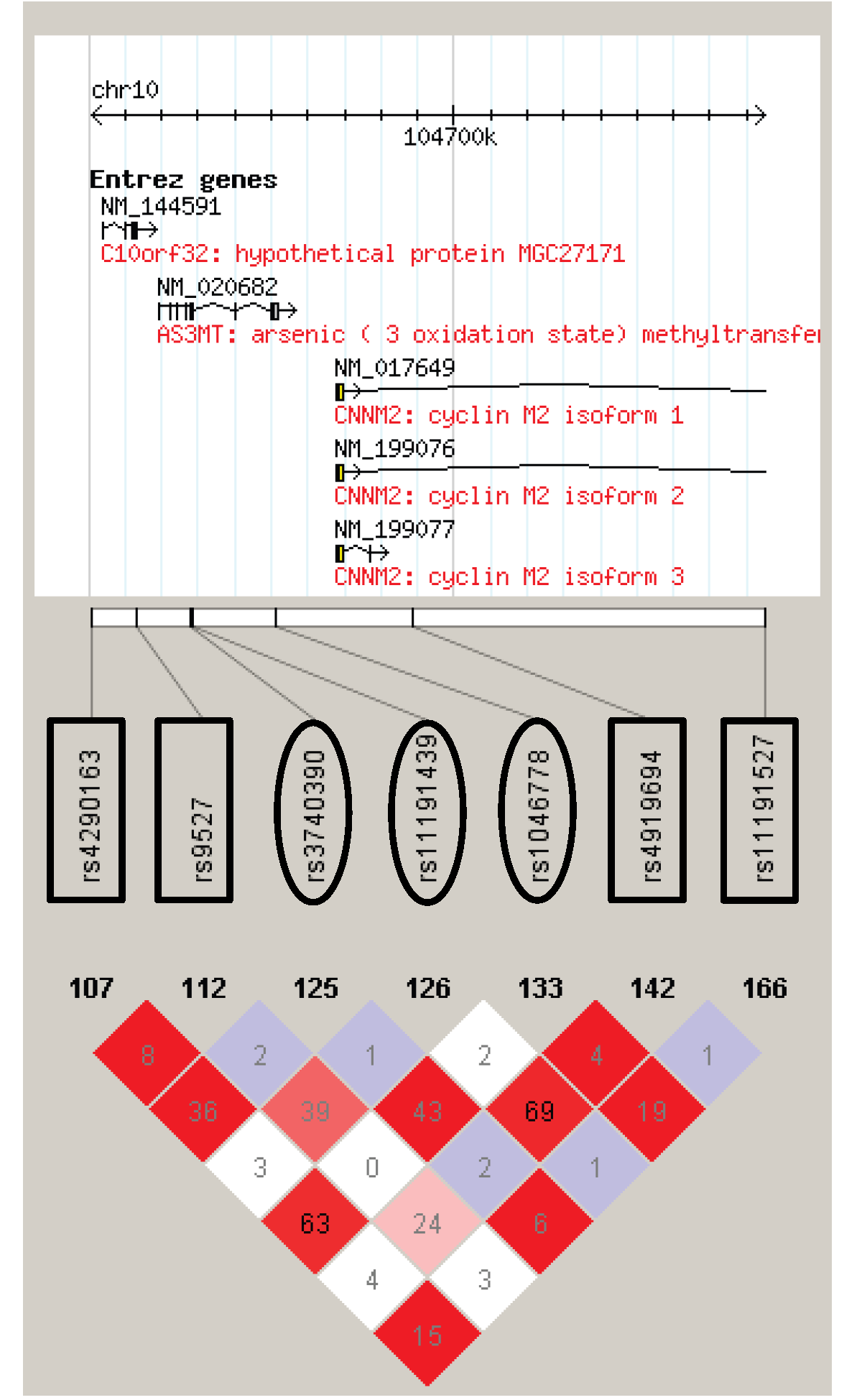

Supplement: Figure S10 — Linkage Disequilibrium between lead SNPs and previously reported variants. Our lead SNPs are shown in boxes and the variants representing the signals previously reported in a Bangladeshi study (Engstrom et al. [30]) are shown in circles. (TIF) [file pgen.1002522.s010.tif]

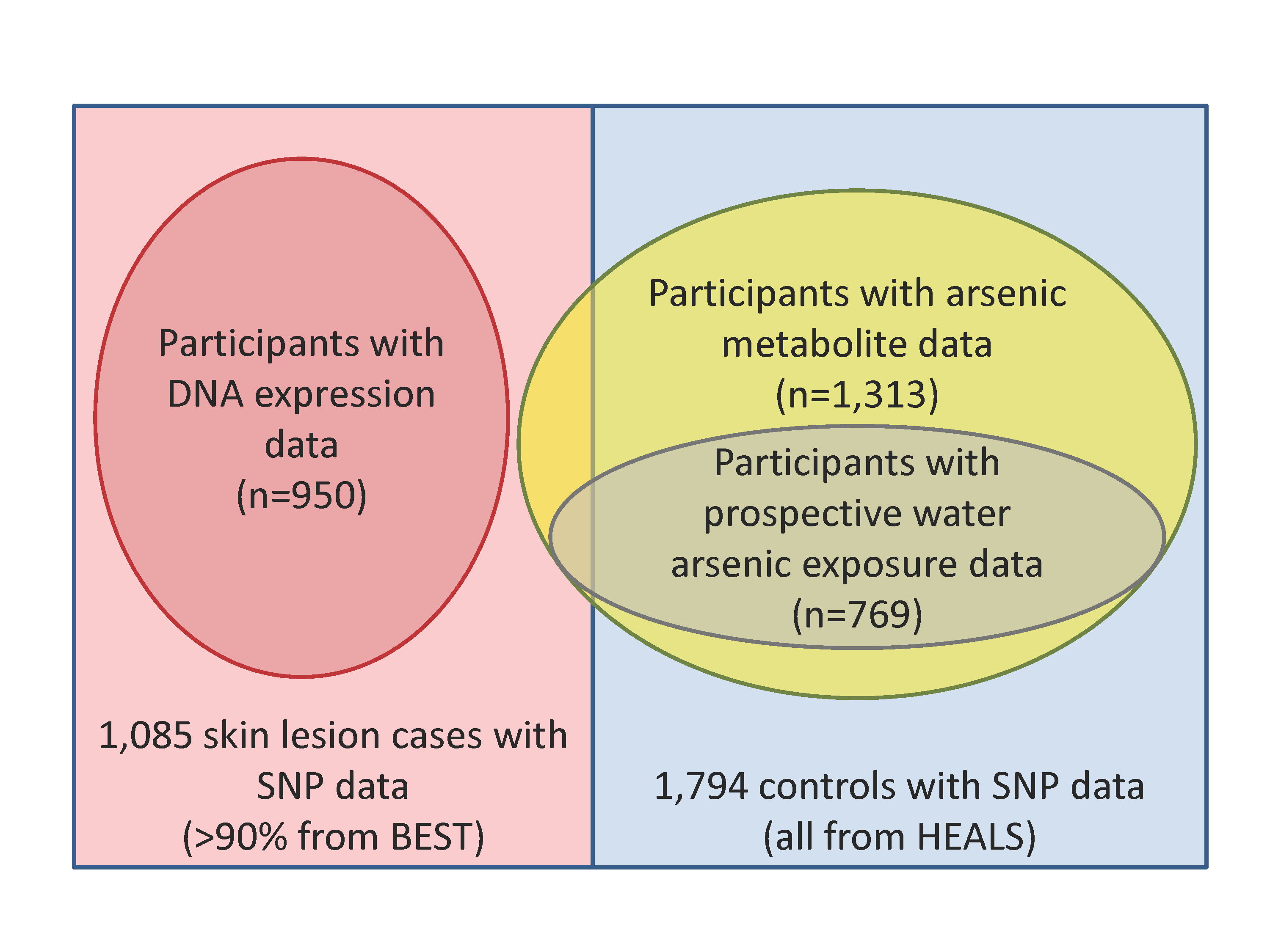

Supplement: Figure S11 — An overview of the participants and samples used in this work. (TIF) [file pgen.1002522.s011.tif]

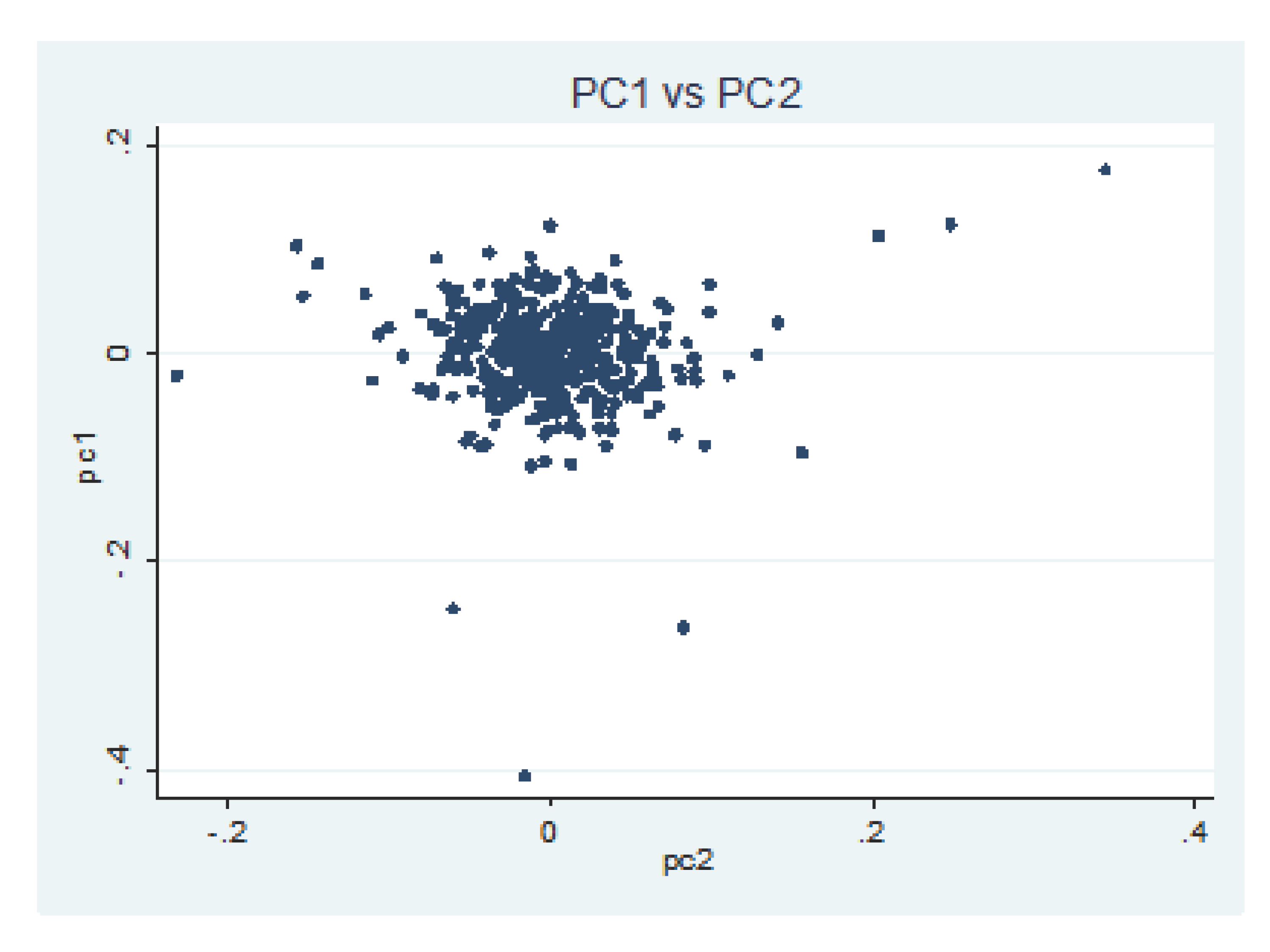

Supplement: Figure S12 — Scatter plot of the first two principle components for 403 unrelated study participants (no pair-wise kinship value>0.05). (TIF) [file pgen.1002522.s012.tif]
