# Supplementary material for: Genome-Wide Association Study Identifies Chromosome 10q24.32 Variants Associated with Arsenic Metabolism and Toxicity Phenotypes in Bangladesh
Source: PLoS Genet. 2012 Feb 23;8(2):e1002522. doi: 10.1371/journal.pgen.1002522 (PMC3285587; doi:10.1371/journal.pgen.1002522)
Supplement: Table S1 — Pair-wise correlations among the arsenic-related urinary phenotypes examined in this study (n = 1,333). (DOCX) [file pgen.1002522.s013.docx]

**Table S1. Pair-wise correlations among the arsenic-related urinary phenotypes examined in this study (n=1,333)**

|  | **MMA%** | **DMA%** | **iAs%** | **Total Urinary As^a^** | **DMA%/MMA%^a^** | **MMA%/iAs%^a^** |
| --- | --- | --- | --- | --- | --- | --- |
| **MMA%** | 1.0 | -0.58 | 0.13 | 0.08 | -0.95 | 0.60 |
| **DMA%** | -0.58 | 1.0 | -0.74 | -0.12 | 0.73 | 0.12 |
| **iAs%** | 0.13 | -0.74 | 1.0 | 0.19 | -0.31 | -0.64 |
| **Total Urinary As^a,b^** | 0.08 | -0.12 | 0.19 | 1.0 | -0.08 | -0.10 |
| **DMA%/MMA%^a^** | -0.95 | 0.73 | -0.31 | -0.08 | 1.0 | -0.49 |
| **MMA%/iAs%^a^** | 0.60 | 0.12 | -0.64 | -0.10 | -0.49 | 1.0 |

All P-values are <0.01.

^a^ Log-transformed

^b^ Adjusted for urinary creatinine.
