# Supplementary material for: Genome-Wide Association Study Identifies Chromosome 10q24.32 Variants Associated with Arsenic Metabolism and Toxicity Phenotypes in Bangladesh
Source: PLoS Genet. 2012 Feb 23;8(2):e1002522. doi: 10.1371/journal.pgen.1002522 (PMC3285587; doi:10.1371/journal.pgen.1002522)
Supplement: Table S2 — Regression models for interaction between arsenic exposure and rs9257 in relation to skin lesion risk (69 skin lesion cases, 701 controls). (DOCX) [file pgen.1002522.s014.docx]

**Table S2. Regression models for interaction between arsenic exposure and rs9257 in relation to skin lesion risk (69 skin lesion cases, 701 controls)**

| **SNP** | **Logistic Regression** | | |
| --- | --- | --- | --- |
| Arsenic exposure | **OR** | **95% CI** | **P** |
| **rs9527** |  |  |  |
| ln(water arsenic ^a^) |  |  |  |
| All individuals | 1.34 | 1.13-1.59 | 0.0007 |
| Among GG | 1.21 | 1.02-1.44 | 0.003 |
| *Among AA+AG* | 2.74 | 1.49-5.05 | 0.0002 |
|  | Multiplicative Interaction P=0.01 | | |
|  | Additive Interaction P=0.004 | | |
|  |  |  |  |
| ln(urinary arsenic ^b^) |  |  |  |
| All individuals | 1.79 | 1.31-2.46 | 0.0003 |
| Among GG | 1.60 | 1.13-2.25 | 0.007 |
| *Among AA+AG* | 3.19 | 1.46-7.00 | 0.004 |
|  | Multiplicative Interaction P=0.11 | | |
|  | Additive Interaction P=0.02 | | |
|  |  |  |  |
| **rs11191659** |  |  |  |
| ln(water arsenic ^a^) |  |  |  |
| All individuals | 1.34 | 1.13-1.59 | 0.0007 |
| Among GG | 1.24 | 1.05-1.47 | 0.01 |
| *Among AA+AG* | 4.72 | 1.56-14.22 | 0.006 |
|  | Multiplicative Interaction P=0.02 | | |
|  | Additive Interaction P=0.001 | | |
|  |  |  |  |
| ln(urinary arsenic ^b^) |  |  |  |
| All individuals | 1.78 | 1.30-2.43 | 0.0003 |
| Among GG | 1.50 | 1.08-2.08 | 0.01 |
| *Among AA+AG* | 10.21 | 2.77-37.63 | 0.0005 |
|  | Multiplicative Interaction P=0.005 | | |
|  | Additive Interaction P<0.0001 | | |

Models are adjusted for age, sex, and BMI. The multiplicative interaction P was generated in a logistic regression using a cross-product term for the genotype-arsenic interaction. The additive interaction P was generated using a mixed linear model which accounted for relatedness using a kinship matrix.

^a^ Measured in grams per microliter

^b^ Measured in micro-grams per gram creatinine
