# Supplementary material for: Genome-Wide Association Study Identifies Chromosome 10q24.32 Variants Associated with Arsenic Metabolism and Toxicity Phenotypes in Bangladesh
Source: PLoS Genet. 2012 Feb 23;8(2):e1002522. doi: 10.1371/journal.pgen.1002522 (PMC3285587; doi:10.1371/journal.pgen.1002522)
Supplement: Table S3 — P-values from association test for our 5 lead 10q24.32 SNPs and expression values for all genes in the 10q24.32 LD region (n = 950 individuals). (DOCX) [file pgen.1002522.s015.docx]

**Table S3. P-values from association test for our 5 lead 10q24.32 SNPs and expression values for all genes in the 10q24.32 LD region (n=950 individuals)**

| **Genes** | **rs4290163** | **rs9527** | **rs4919694** | **rs11191527** | **rs11191659** |
| --- | --- | --- | --- | --- | --- |
| AS3MT | 2.2916E-05 | 2.66E-05 | 2.52E-06 | 0.03 | 0.08 |
| PSD | 0.68 | 0.24 | 0.80 | 0.83 | 0.19 |
| GBF1 | 0.04 | 0.09 | 0.69 | 0.58 | 0.65 |
| NFKB2 | 0.59 | 0.58 | 0.47 | 0.63 | 0.76 |
| FBXL15 | 0.72 | 0.33 | 0.78 | 0.94 | 0.17 |
| CUEDC2 | 0.11 | 0.48 | 0.76 | 0.47 | 0.23 |
| C10orf95 | 0.60 | 0.44 | 0.54 | 0.41 | 0.96 |
| MIR146B | 0.41 | 0.78 | 0.52 | 0.26 | 0.75 |
| TMEM180 | 0.12 | 0.49 | 0.83 | 0.19 | 0.87 |
| ACTR1A | 0.34 | 0.51 | 0.32 | 0.64 | 0.67 |
| SUFU | 0.18 | 0.16 | 0.15 | 0.12 | 0.71 |
| TRIM8 | 0.10 | 0.63 | 0.70 | 0.03 | 0.41 |
| ARL3 | 0.33 | 0.15 | 0.67 | 1.00 | 0.07 |
| SFXN2 | 0.12 | 0.12 | 0.98 | 0.96 | 0.38 |
| CYP17A1 | 0.39 | 0.30 | 0.90 | 0.24 | 0.99 |
| C10orf26 | 0.00005 | 0.01 | 0.02 | 0.002 | 0.04 |
| C10orf32 | 1.12E-11 | 2.60E-41 | 8.69E-21 | 1.1567E-05 | 1.04E-12 |
| CNNM2 | 0.45 | 0.26 | 0.53 | 0.10 | 0.89 |
| NT5C2 | 0.07 | 0.96 | 0.28 | 0.01 | 0.92 |
| LOC729020 | 0.52 | 0.13 | 0.16 | 0.21 | 0.81 |
| PCGF6 | 0.09 | 0.03 | 0.26 | 0.62 | 0.68 |
| INA | 0.35 | 0.74 | 0.56 | 0.20 | 0.97 |
| TAF5 | 0.28 | 0.97 | 0.88 | 0.88 | 0.53 |
| USMG5 | 0.05 | 1.96E-12 | 2.36E-06 | 1.09E-05 | 3.29E-12 |
| PDCD11 | 0.38 | 0.88 | 0.04 | 0.09 | 0.04 |
| CALHM2 | 0.79 | 0.76 | 0.71 | 0.93 | 0.59 |
| CALHM3 | 0.59 | 0.38 | 0.68 | 0.43 | 0.58 |
| CALHM1 | 0.05 | 0.12 | 0.62 | 0.89 | 0.99 |
| NEURL | 0.13 | 0.19 | 0.98 | 0.21 | 0.34 |
| SH3PXD2A | 0.65 | 1.00 | 0.78 | 0.80 | 0.52 |

Genes with expression values that are associated with our lead SNPs are highlighted in grey. The linear mixed models are adjusted for age and sex.
