# Supplementary material for: Genome-Wide Association Study Identifies Chromosome 10q24.32 Variants Associated with Arsenic Metabolism and Toxicity Phenotypes in Bangladesh
Source: PLoS Genet. 2012 Feb 23;8(2):e1002522. doi: 10.1371/journal.pgen.1002522 (PMC3285587; doi:10.1371/journal.pgen.1002522)
Supplement: Table S4 — Functional information for SNPs in LD with rs9527. (PDF) [file pgen.1002522.s016.pdf]

Table S4. Functional information for SNPs in LD with rs9527

No.	rs	Chromosome	Position	Allele	LDsnp	Pop/LD	TFBS	Splicing(site)	Splicing(ESE or ESS)	Splicing(abolish domain)	miRNA(miRanda)	miRNA(Sanger)	nsSNP	Stop Codon	Polyphen	SNPs3D(svm profile)	SNPs3D(svm structure)	RegPotential	Conservation	Nearby Gene	Distance (bp)	Allele	GIH
1	rs10509760	10	104624097	G/A	rs9527	GIH/0.597	--	--	--	--	--	--	--	--	--	--	--	0	0.102	AS3MT	4897||27549	A	0.955
2	rs11191381	10	104483434	C/T	rs9527	GIH/0.543	--	--	--	--	--	--	--	--	--	--	--	0	0.003	SFXN2	19146||5499	C	0.909
3	rs11191401	10	104563393	A/G	rs9527	GIH/0.692	--	--	--	--	Y	--	--	--	--	--	--	0.075831	0.001	C10orf26	69676||2618	A	0.926
4	rs12241712	10	104398262	A/C	rs9527	GIH/0.440	--	--	--	--	--	--	--	--	--	--	--	0.286948	0.038	TRIM8	4020||9804	C	0.892
5	rs12416687	10	104619001	C/T	rs9527	GIH/1.000	Y	--	--	--	--	--	--	--	--	--	--	0	0	C10orf32||AS3MT	-5041||-199	T	0.926
6	rs12573077	10	104424620	A/C	rs9527	GIH/0.440	--	--	--	--	Y	--	--	--	--	--	--	0.387467	0.003	ARL3	1142||39560	C	0.892
7	rs12775883	10	104475291	A/G	rs9527	GIH/0.471	--	--	--	--	--	--	--	--	--	--	--	NA	0	SFXN2	11003||13642	G	0.898
8	rs1475642	10	104536173	A/G	rs9527	GIH/0.692	--	--	--	--	--	--	--	--	--	--	--	0	0.001	C10orf26	42456||29838	A	0.926
9	rs17784294	10	104469375	A/C	rs9527	GIH/0.471	--	--	--	--	--	--	--	--	--	--	--	0.146476	0	SFXN2	5087||19558	C	0.898
10	rs3740394	10	104624464	G/A	rs9527	GIH/0.597	--	--	--	--	--	--	--	--	--	--	--	0.011213	0	AS3MT	5264||27182	A	0.955
11	rs3850699	10	104404211	G/A	rs9527	GIH/0.440	--	--	--	--	--	--	--	--	--	--	--	NA	0	TRIM8	9969||3855	A	0.892
12	rs4919682	10	104574320	C/T	rs9527	GIH/0.692	--	--	--	--	--	--	--	--	--	--	--	NA	0	C10orf26||CYP17A1	-8309||-5958	C	0.926
13	rs4919685	10	104577352	G/T	rs9527	GIH/0.856	--	--	--	--	--	--	--	--	--	--	--	0	0	C10orf26||CYP17A1	-11341||-2926	G	0.915
14	rs4919686	10	104582239	A/C	rs9527	GIH/0.856	--	--	--	--	--	--	--	--	--	--	--	0.229877	0	CYP17A1	1961||5041	A	0.915
15	rs4919687	10	104585238	A/G	rs9527	GIH/0.700	--	--	--	--	--	--	--	--	--	--	--	0.166795	0	CYP17A1	4960||2042	G	0.898
16	rs4919690	10	104606490	C/T	rs9527	GIH/0.797	--	--	--	--	--	--	--	--	--	--	--	0	0	C10orf32	2481||7470	T	0.908
17	rs7904252	10	104446715	G/T	rs9527	GIH/0.471	--	--	--	--	--	Y	--	--	--	--	--	NA	0	ARL3	23237||17465	G	0.898
18	rs7904396	10	104446828	A/G	rs9527	GIH/0.471	--	--	--	--	--	--	--	--	--	--	--	NA	0	ARL3	23350||17352	G	0.898
19	rs7907503	10	104430040	C/T	rs9527	GIH/0.440	--	--	--	--	--	--	--	--	--	--	--	0	0.009	ARL3	6562||34140	C	0.892
20	rs9527	10	104613568	T/C	rs9527	1	--	--	--	--	Y	Y	--	--	--	--	--	0.233079	0.981	C10orf32	9559||392	C	0.926
