# Supplementary material for: Genome-Wide Association Study Identifies Chromosome 10q24.32 Variants Associated with Arsenic Metabolism and Toxicity Phenotypes in Bangladesh
Source: PLoS Genet. 2012 Feb 23;8(2):e1002522. doi: 10.1371/journal.pgen.1002522 (PMC3285587; doi:10.1371/journal.pgen.1002522)
Supplement: Table S5 — Functional information for SNPs in LD with rs11191527. (PDF) [file pgen.1002522.s017.pdf]

Table S5. Functional information for SNPs in LD with rs11191527

No.	rs	Chromosome	Position	Allele	LDsnp	Pop/LD	TFBS	Splicing(site)	Splicing(ESE or ESS)	Splicing(abolish domain)	miRNA(miRanda)	miRNA(Sanger)	nsSNP	Stop Codon	Polyphen	SNPs3D(svm profile)	SNPs3D(svm structure)	RegPotential	Conservation	Nearby Gene	Distance (bp)	Allele	GIH
1	rs10218853	10	104786777	A/C	rs11191527	GIH/0.636	--	--	--	--	--	--	--	--	--	--	--	0	0	CNNM2	118673||41454	A	0.778
2	rs10786737	10	104847208	A/C	rs11191527	GIH/1.000	--	--	--	--	--	--	--	--	--	--	--	0	0	NT5C2	9306||95798	A	0.847
3	rs10786744	10	104935018	A/C	rs11191527	GIH/0.636	--	--	--	--	--	--	--	--	--	--	--	0	0.003	NT5C2	97116||7988	C	0.778
4	rs10883790	10	104630945	A/C	rs11191527	GIH/0.658	--	--	--	--	--	--	--	--	--	--	--	0	0.944	AS3MT	11745||20701	A	0.784
5	rs10883800	10	104661202	A/C	rs11191527	GIH/0.596	--	--	--	--	--	--	--	--	--	--	--	0	0	AS3MT||CNNM2	-9556||-6902	A	0.767
6	rs10883811	10	104717269	A/G	rs11191527	GIH/0.596	--	--	--	--	--	--	--	--	--	--	--	0	0.001	CNNM2	49165||110962	A	0.767
7	rs10883820	10	104754651	A/C	rs11191527	GIH/0.636	--	--	--	--	--	--	--	--	--	--	--	NA	0	CNNM2	86547||73580	C	0.778
8	rs10883839	10	104910331	A/G	rs11191527	GIH/0.596	--	--	--	--	--	--	--	--	--	--	--	NA	0.185	NT5C2	72429||32675	A	0.767
9	rs10883843	10	104937483	C/G	rs11191527	GIH/0.636	--	--	--	--	--	--	--	--	--	--	--	NA	0	NT5C2	99581||5523	C	0.778
10	rs11191457	10	104651698	C/T	rs11191527	GIH/0.837	--	--	--	--	--	--	--	--	--	--	--	0	0.001	AS3MT||CNNM2	-52||-16406	C	0.833
11	rs11191494	10	104743665	A/C	rs11191527	GIH/0.918	--	--	--	--	--	--	--	--	--	--	--	NA	0.015	CNNM2	75561||84566	C	0.833
12	rs11191506	10	104758624	C/T	rs11191527	GIH/0.847	--	--	--	--	--	--	--	--	--	--	--	0	0	CNNM2	90520||69607	C	0.822
13	rs11191516	10	104767862	A/C	rs11191527	GIH/0.847	--	--	--	--	--	--	--	--	--	--	--	0	0.008	CNNM2	99758||60369	C	0.824
14	rs11191527	10	104785124	C/T	rs11191527	1	--	--	--	--	--	--	--	--	--	--	--	0.235806	0.999	CNNM2	117020||43107	C	0.847
15	rs11191549	10	104836787	C/T	rs11191527	GIH/0.636	--	--	--	--	--	--	--	--	--	--	--	0.176759	0	CNNM2||NT5C2	-8556||-1115	C	0.778
16	rs11191554	10	104845268	C/T	rs11191527	GIH/1.000	--	--	--	--	--	--	--	--	--	--	--	0	0.011	NT5C2	7366||97738	C	0.847
17	rs12246739	10	104777009	A/C	rs11191527	GIH/0.636	--	--	--	--	--	--	--	--	--	--	--	0	0.001	CNNM2	108905||51222	C	0.778
18	rs12248123	10	104725356	A/G	rs11191527	GIH/0.541	--	--	--	--	--	--	--	--	--	--	--	0	0	CNNM2	57252||102875	A	0.744
19	rs12573221	10	104839134	A/C	rs11191527	GIH/0.707	--	--	--	--	Y	--	--	--	--	--	--	0.043194	0.004	NT5C2	1232||103872	A	0.886
20	rs12764154	10	104747699	A/C	rs11191527	GIH/0.847	--	--	--	--	--	--	--	--	--	--	--	0	0.494	CNNM2	79595||80532	A	0.824
21	rs12775302	10	104901346	A/G	rs11191527	GIH/0.596	--	--	--	--	--	--	--	--	--	--	--	0	0	NT5C2	63444||41660	A	0.767
22	rs12783467	10	104698302	A/G	rs11191527	GIH/0.785	--	--	--	--	--	--	--	--	--	--	--	0.080959	0	CNNM2	30198||129929	A	0.812
23	rs12785223	10	104834001	A/G	rs11191527	GIH/0.527	--	--	--	--	--	--	--	--	--	--	--	0.141197	0.003	CNNM2||NT5C2	-5770||-3901	A	0.801
24	rs1541213	10	104875320	A/G	rs11191527	GIH/0.596	Y	--	--	--	--	--	--	--	--	--	--	0	0	NT5C2	37418||67686	A	0.767
25	rs1556960	10	104716663	G/A	rs11191527	GIH/0.596	--	--	--	--	--	--	--	--	--	--	--	0.132513	0.002	CNNM2	48559||111568	A	0.767
26	rs17115061	10	104562871	C/T	rs11191527	GIH/0.513	--	--	--	--	--	--	--	--	--	--	--	0.284765	0.233	C10orf26	69154||3140	T	0.915
27	rs17115073	10	104563846	C/T	rs11191527	GIH/0.513	--	--	--	--	Y	--	--	--	--	--	--	0	0.024	C10orf26	70129||2165	C	0.915
28	rs1926029	10	104845660	G/A	rs11191527	GIH/0.636	--	--	--	--	--	--	--	--	--	--	--	0	0.001	NT5C2	7758||97346	G	0.778
29	rs1935323	10	104867025	T/C	rs11191527	GIH/0.629	--	--	--	--	--	--	--	--	--	--	--	0	0.025	NT5C2	29123||75981	T	0.782
30	rs2148198	10	104905300	A/G	rs11191527	GIH/0.596	--	--	--	--	--	--	--	--	--	--	--	0	0.004	NT5C2	67398||37706	G	0.767
31	rs3740386	10	104921041	T/C	rs11191527	GIH/0.552	Y	--	--	--	--	--	--	--	--	--	--	0	0.024	NT5C2	83139||21965	C	0.771
32	rs3740392	10	104626845	T/C	rs11191527	GIH/0.658	--	--	--	--	--	--	--	--	--	--	--	0	0.004	AS3MT	7645||24801	T	0.784
33	rs3781281	10	104842638	G/A	rs11191527	GIH/0.596	--	--	--	--	--	--	--	--	--	--	--	0	0	NT5C2	4736||100368	G	0.767
34	rs3781282	10	104842409	G/A	rs11191527	GIH/0.596	--	--	--	--	--	--	--	--	--	--	--	0	0	NT5C2	4507||100597	A	0.767
35	rs3781283	10	104823038	G/A	rs11191527	GIH/0.628	--	--	--	--	--	--	--	--	--	--	--	0	0.001	CNNM2	154934||5193	G	0.898
36	rs3897402	10	104695402	C/T	rs11191527	GIH/0.847	--	--	--	--	--	--	--	--	--	--	--	0	0.001	CNNM2	27298||132829	C	0.824
37	rs7073323	10	104787413	A/C	rs11191527	GIH/0.616	--	--	--	--	--	--	--	--	--	--	--	0	0	CNNM2	119309||40818	A	0.773
38	rs7074395	10	104834918	G/T	rs11191527	GIH/0.527	--	--	--	--	--	--	--	--	--	--	--	0.121518	0.001	CNNM2||NT5C2	-6687||-2984	G	0.801
39	rs7085854	10	104640241	C/T	rs11191527	GIH/0.803	--	--	--	--	--	--	--	--	--	--	--	NA	0.118	AS3MT	21041||11405	T	0.83
40	rs7089680	10	104792061	C/T	rs11191527	GIH/0.616	--	--	--	--	--	--	--	--	--	--	--	0	0.001	CNNM2	123957||36170	T	0.773
41	rs7894588	10	104746020	A/T	rs11191527	GIH/0.636	--	--	--	--	--	--	--	--	--	--	--	0	0.016	CNNM2	77916||82211	A	0.778
42	rs7898770	10	104746626	A/G	rs11191527	GIH/0.636	--	--	--	--	--	--	--	--	--	--	--	0	0.001	CNNM2	78522||81605	G	0.778
43	rs7899622	10	104717323	C/T	rs11191527	GIH/0.596	--	--	--	--	--	--	--	--	--	--	--	0	0	CNNM2	49219||110908	C	0.767
