# Supplementary material for: Genome-Wide Association Study Identifies Chromosome 10q24.32 Variants Associated with Arsenic Metabolism and Toxicity Phenotypes in Bangladesh
Source: PLoS Genet. 2012 Feb 23;8(2):e1002522. doi: 10.1371/journal.pgen.1002522 (PMC3285587; doi:10.1371/journal.pgen.1002522)
Supplement: Table S6 — Functional information for SNPs in LD with rs4919694. (PDF) [file pgen.1002522.s018.pdf]

Table S6. Functional information for SNPs in LD with rs4919694

<table>
<tr><th>No.</th><th>rs</th><th>Chromosome</th><th>Position</th><th>Allele</th><th>LDsnp</th><th>Pop/LD</th><th>TFBS</th><th>Splicing(site)</th><th>Splicing(ESE or ESS)</th><th>Splicing(abolish domain)</th><th>miRNA(miRanda)</th><th>miRNA(Sanger)</th><th>nsSNP</th><th>Stop Codon</th><th>Polyphen</th><th>SNPs3D(svm profile)</th><th>SNPs3D(svm structure)</th><th>RegPotential</th><th>Conservation</th><th>Nearby Gene</th><th>Distance (bp)</th><th>Allele</th><th>GIH</th></tr>
<tr><td>1</td><td>rs10509760</td><td>10</td><td>104624097</td><td>G/A</td><td>rs4919694</td><td>GIH/0.419</td><td>--</td><td>--</td><td>--</td><td>--</td><td>--</td><td>--</td><td>--</td><td>--</td><td>--</td><td>--</td><td>--</td><td>0</td><td>0.102</td><td>AS3MT</td><td>4897||27549</td><td>A</td><td>0.955</td></tr>
<tr><td>2</td><td>rs11191439</td><td>10</td><td>104628713</td><td>C/T</td><td>rs4919694</td><td>GIH/0.694</td><td>--</td><td>--</td><td>Y</td><td>Y</td><td>--</td><td>--</td><td>Y</td><td>--</td><td>--</td><td>--</td><td>--</td><td>0.183361</td><td>1</td><td>AS3MT</td><td>9513||22933</td><td>T</td><td>0.949</td></tr>
<tr><td>3</td><td>rs11191545</td><td>10</td><td>104819783</td><td>A/G</td><td>rs4919694</td><td>GIH/0.790</td><td>--</td><td>--</td><td>--</td><td>--</td><td>--</td><td>--</td><td>--</td><td>--</td><td>--</td><td>--</td><td>--</td><td>0.026001</td><td>0</td><td>CNNM2</td><td>151679||8448</td><td>G</td><td>0.955</td></tr>
<tr><td>4</td><td>rs12240508</td><td>10</td><td>104929222</td><td>A/G</td><td>rs4919694</td><td>GIH/0.500</td><td>--</td><td>--</td><td>--</td><td>--</td><td>--</td><td>--</td><td>--</td><td>--</td><td>--</td><td>--</td><td>--</td><td>NA</td><td>0</td><td>NT5C2</td><td>91320||13784</td><td>G</td><td>0.931</td></tr>
<tr><td>5</td><td>rs12257935</td><td>10</td><td>104793052</td><td>A/C</td><td>rs4919694</td><td>GIH/0.790</td><td>--</td><td>--</td><td>--</td><td>--</td><td>--</td><td>--</td><td>--</td><td>--</td><td>--</td><td>--</td><td>--</td><td>0.14869</td><td>0.002</td><td>CNNM2</td><td>124948||35179</td><td>C</td><td>0.955</td></tr>
<tr><td>6</td><td>rs3740394</td><td>10</td><td>104624464</td><td>G/A</td><td>rs4919694</td><td>GIH/0.419</td><td>--</td><td>--</td><td>--</td><td>--</td><td>--</td><td>--</td><td>--</td><td>--</td><td>--</td><td>--</td><td>--</td><td>0.011213</td><td>0</td><td>AS3MT</td><td>5264||27182</td><td>A</td><td>0.955</td></tr>
<tr><td>7</td><td>rs4917995</td><td>10</td><td>104835433</td><td>A/T</td><td>rs4919694</td><td>GIH/0.501</td><td>--</td><td>--</td><td>--</td><td>--</td><td>--</td><td>--</td><td>--</td><td>--</td><td>--</td><td>--</td><td>--</td><td>0</td><td>0</td><td>CNNM2||NT5C2</td><td>-7202||-2469</td><td>T</td><td>0.932</td></tr>
<tr><td>8</td><td>rs4919694</td><td>10</td><td>104688968</td><td>C/T</td><td>rs4919694</td><td>1</td><td>--</td><td>--</td><td>--</td><td>--</td><td>--</td><td>--</td><td>--</td><td>--</td><td>--</td><td>--</td><td>--</td><td>0.077234</td><td>0.005</td><td>CNNM2</td><td>20864||139263</td><td>T</td><td>0.943</td></tr>
<tr><td>9</td><td>rs7100592</td><td>10</td><td>104759088</td><td>G/T</td><td>rs4919694</td><td>GIH/0.790</td><td>--</td><td>--</td><td>--</td><td>--</td><td>--</td><td>--</td><td>--</td><td>--</td><td>--</td><td>--</td><td>--</td><td>0</td><td>0.025</td><td>CNNM2</td><td>90984||69143</td><td>T</td><td>0.955</td></tr>
</table>
