# Supplementary material for: Genome-Wide Association Study Identifies Chromosome 10q24.32 Variants Associated with Arsenic Metabolism and Toxicity Phenotypes in Bangladesh
Source: PLoS Genet. 2012 Feb 23;8(2):e1002522. doi: 10.1371/journal.pgen.1002522 (PMC3285587; doi:10.1371/journal.pgen.1002522)
Supplement: Table S7 — Functional information for SNPs in LD with rs3740394. (PDF) [file pgen.1002522.s019.pdf]

Table S7. Functional information for SNPs in LD with rs3740394

<table>
<tr><th>No.</th><th>rs</th><th>Chromosome</th><th>Position</th><th>Allele</th><th>LDsnp</th><th>Pop/LD</th><th>TFBS</th><th>Splicing(site)</th><th>Splicing(ESE or ESS)</th><th>Splicing(abolish domain)</th><th>miRNA(miRanda)</th><th>miRNA(Sanger)</th><th>nsSNP</th><th>Stop Codon</th><th>Polyphen</th><th>SNPs3D(svm profile)</th><th>SNPs3D(svm structure)</th><th>RegPotential</th><th>Conservation</th><th>Nearby Gene</th><th>Distance (bp)</th><th>Allele</th><th>GIH</th></tr>
<tr><td>1</td><td>rs10509760</td><td>10</td><td>104624097</td><td>G/A</td><td>rs3740394</td><td>GIH/1.000</td><td>--</td><td>--</td><td>--</td><td>--</td><td>--</td><td>--</td><td>--</td><td>--</td><td>--</td><td>--</td><td>--</td><td>0</td><td>0.102</td><td>AS3MT</td><td>4897||27549</td><td>A</td><td>0.955</td></tr>
<tr><td>2</td><td>rs11191381</td><td>10</td><td>104483434</td><td>C/T</td><td>rs3740394</td><td>GIH/0.476</td><td>--</td><td>--</td><td>--</td><td>--</td><td>--</td><td>--</td><td>--</td><td>--</td><td>--</td><td>--</td><td>--</td><td>0</td><td>0.003</td><td>SFXN2</td><td>19146||5499</td><td>C</td><td>0.909</td></tr>
<tr><td>3</td><td>rs11191401</td><td>10</td><td>104563393</td><td>A/G</td><td>rs3740394</td><td>GIH/0.597</td><td>--</td><td>--</td><td>--</td><td>--</td><td>Y</td><td>--</td><td>--</td><td>--</td><td>--</td><td>--</td><td>--</td><td>0.075831</td><td>0.001</td><td>C10orf26</td><td>69676||2618</td><td>A</td><td>0.926</td></tr>
<tr><td>4</td><td>rs11191439</td><td>10</td><td>104628713</td><td>C/T</td><td>rs3740394</td><td>GIH/0.664</td><td>--</td><td>--</td><td>Y</td><td>Y</td><td>--</td><td>--</td><td>Y</td><td>--</td><td>--</td><td>--</td><td>--</td><td>0.183361</td><td>1</td><td>AS3MT</td><td>9513||22933</td><td>T</td><td>0.949</td></tr>
<tr><td>5</td><td>rs11191545</td><td>10</td><td>104819783</td><td>A/G</td><td>rs3740394</td><td>GIH/0.540</td><td>--</td><td>--</td><td>--</td><td>--</td><td>--</td><td>--</td><td>--</td><td>--</td><td>--</td><td>--</td><td>--</td><td>0.026001</td><td>0</td><td>CNNM2</td><td>151679||8448</td><td>G</td><td>0.955</td></tr>
<tr><td>6</td><td>rs12257935</td><td>10</td><td>104793052</td><td>A/C</td><td>rs3740394</td><td>GIH/0.540</td><td>--</td><td>--</td><td>--</td><td>--</td><td>--</td><td>--</td><td>--</td><td>--</td><td>--</td><td>--</td><td>--</td><td>0.14869</td><td>0.002</td><td>CNNM2</td><td>124948||35179</td><td>C</td><td>0.955</td></tr>
<tr><td>7</td><td>rs12416687</td><td>10</td><td>104619001</td><td>C/T</td><td>rs3740394</td><td>GIH/0.597</td><td>Y</td><td>--</td><td>--</td><td>--</td><td>--</td><td>--</td><td>--</td><td>--</td><td>--</td><td>--</td><td>--</td><td>0</td><td>0</td><td>C10orf32||AS3MT</td><td>-5041||-199</td><td>T</td><td>0.926</td></tr>
<tr><td>8</td><td>rs12775883</td><td>10</td><td>104475291</td><td>A/G</td><td>rs3740394</td><td>GIH/0.418</td><td>--</td><td>--</td><td>--</td><td>--</td><td>--</td><td>--</td><td>--</td><td>--</td><td>--</td><td>--</td><td>--</td><td>NA</td><td>0</td><td>SFXN2</td><td>11003||13642</td><td>G</td><td>0.898</td></tr>
<tr><td>9</td><td>rs1475642</td><td>10</td><td>104536173</td><td>A/G</td><td>rs3740394</td><td>GIH/0.597</td><td>--</td><td>--</td><td>--</td><td>--</td><td>--</td><td>--</td><td>--</td><td>--</td><td>--</td><td>--</td><td>--</td><td>0</td><td>0.001</td><td>C10orf26</td><td>42456||29838</td><td>A</td><td>0.926</td></tr>
<tr><td>10</td><td>rs17784294</td><td>10</td><td>104469375</td><td>A/C</td><td>rs3740394</td><td>GIH/0.418</td><td>--</td><td>--</td><td>--</td><td>--</td><td>--</td><td>--</td><td>--</td><td>--</td><td>--</td><td>--</td><td>--</td><td>0.146476</td><td>0</td><td>SFXN2</td><td>5087||19558</td><td>C</td><td>0.898</td></tr>
<tr><td>11</td><td>rs3740394</td><td>10</td><td>104624464</td><td>G/A</td><td>rs3740394</td><td>1</td><td>--</td><td>--</td><td>--</td><td>--</td><td>--</td><td>--</td><td>--</td><td>--</td><td>--</td><td>--</td><td>--</td><td>0.011213</td><td>0</td><td>AS3MT</td><td>5264||27182</td><td>A</td><td>0.955</td></tr>
<tr><td>12</td><td>rs4919682</td><td>10</td><td>104574320</td><td>C/T</td><td>rs3740394</td><td>GIH/0.597</td><td>--</td><td>--</td><td>--</td><td>--</td><td>--</td><td>--</td><td>--</td><td>--</td><td>--</td><td>--</td><td>--</td><td>NA</td><td>0</td><td>C10orf26||CYP17A1</td><td>-8309||-5958</td><td>C</td><td>0.926</td></tr>
<tr><td>13</td><td>rs4919685</td><td>10</td><td>104577352</td><td>G/T</td><td>rs3740394</td><td>GIH/0.511</td><td>--</td><td>--</td><td>--</td><td>--</td><td>--</td><td>--</td><td>--</td><td>--</td><td>--</td><td>--</td><td>--</td><td>0</td><td>0</td><td>C10orf26||CYP17A1</td><td>-11341||-2926</td><td>G</td><td>0.915</td></tr>
<tr><td>14</td><td>rs4919686</td><td>10</td><td>104582239</td><td>A/C</td><td>rs3740394</td><td>GIH/0.511</td><td>--</td><td>--</td><td>--</td><td>--</td><td>--</td><td>--</td><td>--</td><td>--</td><td>--</td><td>--</td><td>--</td><td>0.229877</td><td>0</td><td>CYP17A1</td><td>1961||5041</td><td>A</td><td>0.915</td></tr>
<tr><td>15</td><td>rs4919687</td><td>10</td><td>104585238</td><td>A/G</td><td>rs3740394</td><td>GIH/0.418</td><td>--</td><td>--</td><td>--</td><td>--</td><td>--</td><td>--</td><td>--</td><td>--</td><td>--</td><td>--</td><td>--</td><td>0.166795</td><td>0</td><td>CYP17A1</td><td>4960||2042</td><td>G</td><td>0.898</td></tr>
<tr><td>16</td><td>rs4919690</td><td>10</td><td>104606490</td><td>C/T</td><td>rs3740394</td><td>GIH/0.476</td><td>--</td><td>--</td><td>--</td><td>--</td><td>--</td><td>--</td><td>--</td><td>--</td><td>--</td><td>--</td><td>--</td><td>0</td><td>0</td><td>C10orf32</td><td>2481||7470</td><td>T</td><td>0.908</td></tr>
<tr><td>17</td><td>rs4919694</td><td>10</td><td>104688968</td><td>C/T</td><td>rs3740394</td><td>GIH/0.419</td><td>--</td><td>--</td><td>--</td><td>--</td><td>--</td><td>--</td><td>--</td><td>--</td><td>--</td><td>--</td><td>--</td><td>0.077234</td><td>0.005</td><td>CNNM2</td><td>20864||139263</td><td>T</td><td>0.943</td></tr>
<tr><td>18</td><td>rs7100592</td><td>10</td><td>104759088</td><td>G/T</td><td>rs3740394</td><td>GIH/0.540</td><td>--</td><td>--</td><td>--</td><td>--</td><td>--</td><td>--</td><td>--</td><td>--</td><td>--</td><td>--</td><td>--</td><td>0</td><td>0.025</td><td>CNNM2</td><td>90984||69143</td><td>T</td><td>0.955</td></tr>
<tr><td>19</td><td>rs7904252</td><td>10</td><td>104446715</td><td>G/T</td><td>rs3740394</td><td>GIH/0.418</td><td>--</td><td>--</td><td>--</td><td>--</td><td>--</td><td>Y</td><td>--</td><td>--</td><td>--</td><td>--</td><td>--</td><td>NA</td><td>0</td><td>ARL3</td><td>23237||17465</td><td>G</td><td>0.898</td></tr>
<tr><td>20</td><td>rs7904396</td><td>10</td><td>104446828</td><td>A/G</td><td>rs3740394</td><td>GIH/0.418</td><td>--</td><td>--</td><td>--</td><td>--</td><td>--</td><td>--</td><td>--</td><td>--</td><td>--</td><td>--</td><td>--</td><td>NA</td><td>0</td><td>ARL3</td><td>23350||17352</td><td>G</td><td>0.898</td></tr>
<tr><td>21</td><td>rs9527</td><td>10</td><td>104613568</td><td>T/C</td><td>rs3740394</td><td>GIH/0.597</td><td>--</td><td>--</td><td>--</td><td>--</td><td>Y</td><td>Y</td><td>--</td><td>--</td><td>--</td><td>--</td><td>--</td><td>0.233079</td><td>0.981</td><td>C10orf32</td><td>9559||392</td><td>C</td><td>0.926</td></tr>
</table>
