# Supplementary material for: Genome-Wide Association Study Identifies Chromosome 10q24.32 Variants Associated with Arsenic Metabolism and Toxicity Phenotypes in Bangladesh
Source: PLoS Genet. 2012 Feb 23;8(2):e1002522. doi: 10.1371/journal.pgen.1002522 (PMC3285587; doi:10.1371/journal.pgen.1002522)
Supplement: Table S8 — Functional information for SNPs in LD with rs11191659. (PDF) [file pgen.1002522.s020.pdf]

Table S8. Functional information for SNPs in LD with rs11191659

<table>
<tr><th>No.</th><th>rs</th><th>Chromosome</th><th>Position</th><th>Allele</th><th>LDsnp</th><th>Pop/LD</th><th>TFBS</th><th>Splicing(site)</th><th>Splicing(ESE or ESS)</th><th>Splicing(abolish domain)</th><th>miRNA(miRanda)</th><th>miRNA(Sanger)</th><th>nsSNP</th><th>Stop Codon</th><th>Polyphen</th><th>SNPs3D(svm profile)</th><th>SNPs3D(svm structure)</th><th>RegPotential</th><th>Conservation</th><th>Nearby Gene</th><th>Distance (bp)</th><th>Allele</th><th>GIH</th></tr>
<tr><td>1</td><td>rs10883841</td><td>10</td><td>104924699</td><td>C/T</td><td>rs11191659</td><td>GIH/0.828</td><td>--</td><td>--</td><td>Y</td><td>--</td><td>--</td><td>--</td><td>Y</td><td>--</td><td>benign</td><td>--</td><td>--</td><td>0.17821</td><td>1</td><td>NT5C2</td><td>86797||18307</td><td>T</td><td>0.972</td></tr>
<tr><td>2</td><td>rs11191606</td><td>10</td><td>104946025</td><td>C/T</td><td>rs11191659</td><td>GIH/1.000</td><td>Y</td><td>--</td><td>--</td><td>--</td><td>--</td><td>--</td><td>--</td><td>--</td><td>--</td><td>--</td><td>--</td><td>0</td><td>0.02</td><td>LOC729081||LOC401648</td><td>-19983||-19431</td><td>T</td><td>0.966</td></tr>
<tr><td>3</td><td>rs11191655</td><td>10</td><td>105068783</td><td>C/T</td><td>rs11191659</td><td>GIH/1.000</td><td>--</td><td>--</td><td>--</td><td>--</td><td>--</td><td>--</td><td>--</td><td>--</td><td>--</td><td>--</td><td>--</td><td>0</td><td>0.014</td><td>PCGF6</td><td>16240||32098</td><td>T</td><td>0.966</td></tr>
<tr><td>4</td><td>rs11191659</td><td>10</td><td>105091691</td><td>C/T</td><td>rs11191659</td><td>1</td><td>--</td><td>--</td><td>--</td><td>--</td><td>--</td><td>--</td><td>--</td><td>--</td><td>--</td><td>--</td><td>--</td><td>NA</td><td>0</td><td>PCGF6</td><td>39148||9190</td><td>C</td><td>0.966</td></tr>
</table>
